# A step into the rare biosphere: genomic features of the new genus *Terrihalobacillus* and the new species *Aquibacillus salsiterrae* from hypersaline soils

**DOI:** 10.3389/fmicb.2023.1192059

**Published:** 2023-05-09

**Authors:** Cristina Galisteo, Rafael R. de la Haba, Cristina Sánchez-Porro, Antonio Ventosa

**Affiliations:** Department of Microbiology and Parasitology, Faculty of Pharmacy, University of Sevilla, Sevilla, Spain

**Keywords:** *Terrihalobacillus*, *Aquibacillus*, *Bacillota*, hypersaline soils, osmoregulation mechanism, phylogenomics, genome mining, rare biosphere

## Abstract

Hypersaline soils are a source of prokaryotic diversity that has been overlooked until very recently. The phylum *Bacillota*, which includes the genus *Aquibacillus*, is one of the 26 phyla that inhabit the heavy metal contaminated soils of the Odiel Saltmarshers Natural Area (Southwest Spain), according to previous research. In this study, we isolated a total of 32 strains closely related to the genus *Aquibacillus* by the traditional dilution-plating technique. Phylogenetic studies clustered them into two groups, and comparative genomic analyses revealed that one of them represents a new species within the genus *Aquibacillus*, whereas the other cluster constitutes a novel genus of the family *Bacillaceae*. We propose the designations *Aquibacillus salsiterrae* sp. nov. and *Terrihalobacillus insolitus* gen. nov., sp. nov., respectively, for these two new taxa. Genome mining analysis revealed dissimilitude in the metabolic traits of the isolates and their closest related genera, remarkably the distinctive presence of the well-conserved pathway for the biosynthesis of molybdenum cofactor in the species of the genera *Aquibacillus* and *Terrihalobacillus*, along with genes that encode molybdoenzymes and molybdate transporters, scarcely found in metagenomic dataset from this area. In-silico studies of the osmoregulatory strategy revealed a *salt-out* mechanism in the new species, which harbor the genes for biosynthesis and transport of the compatible solutes ectoine and glycine betaine. Comparative genomics showed genes related to heavy metal resistance, which seem required due to the contamination in the sampling area. The low values in the genome recruitment analysis indicate that the new species of the two genera, *Terrihalobacillus* and *Aquibacillus*, belong to the rare biosphere of representative hypersaline environments.

## 1. Introduction

The genus *Aquibacillus,* first described in 2014, is one of the more than 100 genera of the family *Bacillaceae* within the phylum *Bacillota*. At the time of writing, it comprises a total of seven species (Parte et al., 2020), two of them being a reclassification of previously described *Virgibacillus* species (Amoozegar et al., 2014): *Aquibacillus halophilus* (Amoozegar et al., 2014), *Aquibacillus koreensis* (Lee et al., 2006; Amoozegar et al., 2014), *Aquibacillus albus* (Zhang et al., 2012; Amoozegar et al., 2014), *Aquibacillus salifodinae* (Zhang et al., 2015), *Aquibacillus sediminis* (Lee and Whang, 2019), *Aquibacillus kalidii* (Wang et al., 2021), and *Aquibacillus saliphilus* (Cho and Whang, 2022). These species have been isolated from hypersaline environments, such as salt mine (Zhang et al., 2015), saltern soils (Lee et al., 2006; Lee and Whang, 2019), salt lakes (Zhang et al., 2012; Amoozegar et al., 2014), grey salterns (Cho and Whang, 2022), and *Kalidium cuspidatum* plants from saltern lands (Wang et al., 2021). The moderately halophilic species of the genus *Aquibacillus* are Gram-stain-positive endospore-forming rods with optimum growth between 4 and 10% (w/v) NaCl, at pH 7–8, and 25–37°C. They are motile and strictly aerobic, although the species *A. saliphilus* presents a facultatively anaerobic metabolism (Cho and Whang, 2022). The pigmentation of the colonies is cream to white color, their major fatty acid is anteiso-C_15:0_ and their most predominant polar lipids are phosphatidylglycerol and diphosphatidylglycerol (Lee et al., 2006; Zhang et al., 2012, 2015; Amoozegar et al., 2014; Lee and Whang, 2019; Wang et al., 2021; Cho and Whang, 2022). In 2019, a new species with a very close relationship with the genus *Aquibacillus* was described as a new genus, *Radiobacillus*, based on its lack of motility and the presence of an aminophospholipid as one of the major polar lipids, among other characteristics (Li et al., 2020).

The Odiel Saltmarshes Natural Area represents a saline environment in Huelva, Southwest Spain, specifically between the Odiel and Tinto rivers. The area has suffered from industrial and mining activity for years, and some studies have determined it as contaminated by heavy metals (i.e., arsenic, cadmium, copper, lead, and zinc) (Sainz et al., 2002, 2004). The prokaryotic diversity of its hypersaline soils has been previously studied by metagenomic techniques (Vera-Gargallo and Ventosa, 2018; Vera-Gargallo et al., 2019), which detected the phylum *Bacillota* as a minor fraction among the 26 different retrieved phyla. To our best knowledge, there are no reference studies on the ecological distribution of *Aquibacillus* other than its presence in table salt, determined by metataxonomic and culturomic approaches (Satari et al., 2021).

The present study reports the isolation and characterization of 32 novel strains closely related to the genus *Aquibacillus*, within the family *Bacillaceae*. In order to determine their taxonomic position, an in-depth phylogenomic analysis of three selected strains was carried out along with supporting phylogenetic, chemotaxonomic, and phenotypic comparative studies. Additionally, we investigated the functional annotation of the genomes to perceive similarities and differences between the isolates and several genera of the family *Bacillaceae*, in particular, *Aquibacillus*, *Radiobacillus*, and *Sediminibacillus.* Besides, we further dug into the genome sequences to unveil possible mechanisms of adaptation of the isolates to the extreme habitat where they inhabit (i.e., osmoregulation and heavy metal resistance strategies).

## 2. Materials and methods

### 2.1. Odiel Saltmarshes Natural Area sampling and isolation of strains

Sampling on the hypersaline soils located at the saltmarshes of the Odiel Natural Area, in Huelva, Southwest Spain (37°12′26.6″N 6°57′52.5″W), was carried out in Whirl-Pak bags as indicated by Vera-Gargallo et al. (2019). The pH, electrical conductivity, and the concentration of arsenic, cadmium, copper, lead, and zinc were measured as described by Galisteo et al. (2023). Dilution-plating technique was used for the isolation of the strains on R2A medium supplemented with 7.5% (w/v) salts, after 3 months of incubation at 28°C, and then the colonies were subcultured on the same medium until pure cultures were obtained. The composition of the R2A medium is (g L^−1^): yeast extract, 0.5; proteose peptone no. 3, 0.5; casamino acids, 0.5; dextrose, 0.5; starch, 0.5; sodium pyruvate, 0.3; K_2_HPO_4_, 0.3; MgSO_4_, 0.05. This medium was supplemented with a concentrated seawater (SW) stock diluted to a final salt concentration of 7.5% (w/v), and the pH was adjusted to 7.5. For solid medium, commercial R2A agar (Difco) was prepared with the aforementioned pH and salt concentration and supplemented with 2.0% (w/v) agar. The composition of the SW stock was the following: (g L^−1^): NaCl, 234.0; MgCl_2_·6H_2_O, 39.0; MgSO_4_·7H_2_O, 61.0; CaCl_2_, 1.0; KCl, 6.0; NaHCO_3_, 0.2; NaBr, 0.7. For long-term preservation, the liquid culture was mixed with 40% (v/v) glycerol and stored at -80°C. Besides, Marine Agar (MA, Difco 2216) supplemented with 6% (w/v) NaCl was prepared for better comparative purposes of the fatty acid composition with species of the genera *Aquibacillus* and *Radiobacillus*. The following type strains of the genus *Aquibacillus* were obtained from culture collections and used as reference strains for phenotypic comparative analysis: *A. albus* JCM 17364^T^, *A. koreensis* JCM 12387^T^, and *A. salifodinae* JCM 19761^T^. The same medium and conditions stated above were used for their routinary growth. The genomic material of strain *A. koreensis* JCM 12387^T^ was extracted, purified, and sequenced, as explained below, for phylogenomic comparative purposes.

### 2.2. Phylogenetic analyses

The method of Marmur (1961) modified for small volumes was used for genomic DNA extraction of the isolates. The universal primers used for 16S rRNA gene amplification were 27F (5′-AGA GTT TGA TCM TGG CTC AG-3′) and 1492R (5′-GGT TAC CTT GTT ACG ACT T-3′) (Lane, 1991). The PCR product was sequenced using Sanger methodology by StabVida (Caparica, Portugal). Library preparation of genomic material from strains 3ASR75-54^T^, 3ASR75-11^T^, and 3ASR75-286 was performed using Novogene NGS DNA Library Prep Set (Cat. No. PT004), followed by whole shotgun sequencing of the genomes on an Illumina NovaSeq PE150 platform by Novogene Europe (Cambridge, United Kingdom). The same protocol was carried out for *A. koreensis* JCM 12387^T^, whose genome was not previously available.

Identification of the new isolates was achieved by comparing their partial or almost complete 16S rRNA gene sequences against the EzBioCloud database for prokaryotes^1^ (Yoon et al., 2017). The identity shared among the strains isolated in this study was calculated by BLASTn v2.2.28+.^2^ For phylogenetic tree reconstructions, the 16S rRNA gene sequences from the closest related species to the new strains were obtained from SILVA (Quast et al., 2013) and GenBank databases (Clark et al., 2016). The fast aligner tool integrated in the ARB package (Ludwig et al., 2004) was employed to align the sequences at the primary and secondary structure level. Maximum-likelihood (Felsenstein, 1981), maximum-parsimony (Felsenstein, 1983), and neighbor-joining (Saitou and Nei, 1987) algorithms, implemented in the ARB package software (Ludwig et al., 2004), were used for tree inferences, and the Jukes-Cantor was selected as the nucleotide substitution model (Jukes and Cantor, 1969) to correct the distance matrix. Bootstrap analysis with 1,000 pseudoreplicates was carried out in order to validate the robustness of the branches. The script “gitana”^3^ performed the visual editing of the tree.

### 2.3. Comparative genome analyses and ecological distribution

SPAdes v3.15.2 (Prjibelski et al., 2020) was utilized to assemble the quality filtered paired-end reads (options “--careful -k 21, 33, 55, 77, 99, 127”). Contigs shorter than 500 bp or SPAdes coverage below 20 were removed. QUAST v2.3 (Gurevich et al., 2013) allowed us to calculate the assembly statistics and CheckM v1.0.5 (Parks et al., 2015) to evaluate the completeness and contamination of the assembled genomes. In order to sort the contigs of the draft genomes, they were aligned against the closest related strain with available complete genome, i.e., *Radiobacillus deserti* TKL69^T^, by using nucmer, integrated in MUMmer v4.0.0rc1 compilation of utilities and scripts (Marçais et al., 2018). Coding sequences (CDS) were extracted with Prodigal v2.60 (Hyatt et al., 2010) and annotated with Prokka v1.12 (Seemann, 2014) to generate the standard GenBank files. The online tool BlastKOALA (Kanehisa et al., 2016) was employed to perform a detailed functional annotation of the predicted translated CDS, by assigning KEGG Orthology (KO) identifiers and KEGG pathways. The “iep” program from EMBOSS package v6.5.7.0 (Rice et al., 2000) was utilized to determine the isoelectric point of the predicted proteins.

In-depth placement of the three sequenced isolates (strains 3ASR75-54^T^, 3ASR75-11^T^, and 3ASR75-286) within the family *Bacillaceae* was carried out by phylogenomic reconstruction based on the concatenation of the translated single-copy core genes from 79 members of this family whose genome sequence was available in RefSeq database. BLASTp v2.2.28+ and Markov Cluster Algorithm, implemented in the Enveomics toolbox (Rodriguez-R and Konstantinidis, 2016), were used to search and to extract the translated orthologous genes, which were further aligned with Muscle v3.8.31 (Edgar, 2004). FastTreeMP v2.1.8 (Price et al., 2010) was employed to infer the approximately maximum-likelihood phylogeny based on 739 concatenated protein sequences, considering the Jones-Taylor-Thornton model of amino acid evolution (Jones et al., 1992). The robustness of the obtained nodes was checked by the Shimodaira-Hasegawa test (Shimodaira and Hasegawa, 1999). Tree image was edited and visualized with the script “gitana” (see text footnote 3). “UpSetR” v1.4.0 package for R (Conway et al., 2017) allowed us to visualize the intersection of the 18,524 orthologous genes identified after BLASTp search of their translated sequences. The proposed minimal standards for prokaryotic taxonomy (Chun et al., 2018) suggest the use of Overall Genome Relatedness Indexes (OGRIs) for a reliable determination of the taxonomic status of new taxa, such as the digital DNA–DNA hybridization (dDDH), the Average Amino acid Identity (AAI), and the Average Nucleotide Identity for orthologous sequences (orthoANI). The Genome-to-Genome Distance Calculator (GGDC v3.0) from the Leibniz Institute DSMZ (Meier-Kolthoff et al., 2021) was utilized to obtain the dDDH relatedness values, whereas the Enveomics toolbox (Rodriguez-R and Konstantinidis, 2016) and OAU software v1.2 (Lee et al., 2016) were selected for AAI and orthoANI calculations, respectively.

Metagenomic dataset SMO1 (Supplementary Table S1) from a hypersaline soil of the Odiel Saltmarshes Natural Area (Vera-Gargallo et al., 2018) was selected for the screening of functional genes in the environment under study. Raw reads with length ≥ 30 bp were assembled with Megahit v1.2.9 (Li et al., 2015, 2016). Contigs were examined to extract translated CDS by Prodigal v2.60 (Hyatt et al., 2010), and KO identifiers were assigned to them by GhostKOALA (Kanehisa et al., 2016). Then, functions of interest were manually selected.

The ecological distribution of the new strains in hypersaline environments was determined by fragment recruitment analysis against 16 environmental metagenomic datasets (Supplementary Table S1). The 16S rRNA gene sequences from the genomes were masked due to their highly conserved nature. Metagenomic reads above ≥30 bp were BLASTn v2.2.28+ searched, independently, against each genome. BLASTn results with identity values <95%, alignment length < 50 bp, and e-value >10^-5^ were filtered out, as recommended by Mehrshad et al. (2018). In order to normalize the relative abundance values, we computed the RPKG (reads recruited per kilobase of genome per gigabase of metagenome), proposed by Nayfach and Pollard (2015). Besides, the genomes of *Haloquadratum walsbyi* C23^T^ (GCF_000237865.1), *Salinibacter ruber* DSM 13855^T^ (GCF_000013045.1), and *Spiribacter salinus* M19-40^T^ (GCF_000319575.2) were included in the analysis as references for comparison.

Plots generated in this study were created with the following R packages: “aplot” v0.1.8 (Guangchuang et al., 2022), “gghighlight” v0.3.2 (Yutani, 2021), “ggplot2” v3.3.3 (Wickham, 2009), “ggpubr” v0.4.0 (Kassambara, 2020), “ggtext” v0.1.2 (Wilke and Wiernik, 2022), “gridExtra” v2.3 (Auguie, 2017), and “paletteer” v1.4.0 (Hvitfeldt, 2021). R packages “phytools” v1.2-0 (Revell, 2012), “reshape2” v1.4.4 (Wickham, 2007), “scale” v.1.1.1 (Wickham and Seidel, 2020), and “seqinr” v4.2-16 (Charif and Lobry, 2007) were requested to reformat input data. DNAplotter application (Carver et al., 2009) was used to generate the circular representation of the genomes.

### 2.4. Fatty acids composition and phenotypic features

The fatty acid profile of the type strains of the two proposed species, 3ASR75-54^T^ and 3ASR75-11^T^, was determined by gas chromatography with an Agilent 6850 system at the Spanish Type Culture Collection (CECT), Valencia, Spain. For that purpose, strains 3ASR75-54^T^ and 3ASR75-11^T^ were grown in MA medium supplemented with 6% (w/v) NaCl at 35°C for 3 days. TSBA6 library (MIDI, 2008) was used for the determination of fatty acids following the protocol suggested by MIDI Microbial Identification System (Sasser, 1990).

Colonial morphology and pigmentation of strains 3ASR75-54^T^, 3ASR75-11^T^, and 3ASR75-286 were observed after 3 days of growth on R2A medium supplemented with 7.5% (w/v) salts and the pH adjusted to 7.5, at 37°C. Cell morphology and motility were determined by phase contrast microscopy (Olympus CX41). To detect their ability to grow anaerobically, strains 3ASR75-54^T^, 3ASR75-11^T^, and 3ASR75-286 were incubated using the AnaeroGen™ system (Oxoid) under the aforementioned conditions. Optical density measures allowed us to determine the range and optimum salt concentration and pH values supporting growth for type strains 3ASR75-54^T^ and 3ASR75-11^T^. Infinite M Nano microplate reader (Tecan, Grödig, Austria) adjusted at 37°C, with linear shaking, was utilized to measure the absorbance at 600 nm every 2 h for 3 days. R2A broth was supplemented with SW stock to obtain a final salt concentration of 0.5, 2, 4, 5, 6, 7, 7.5, 8, 9, 10, 12, 15, 17, 20, 22, and 25% (w/v) in order to determine the salinity range and optimum. R2A liquid medium supplemented with optimum salt concentration was also employed to test growth at pH values of 3.0, 4.0, 5.0, 6.0, 7.0, 7.5, 8.0, 9.0, and 10.0, using a buffered system to maintain pH conditions (Sánchez-Porro et al., 2009). The temperature range for growth was measured in R2A broth adjusted to the optimal salinity and pH, and incubated at 2, 3, 4, 5, 6, 8, 9, 10, 11, 12, 13, 14, 15, 28, 37, 40, 42, 43, 44, 45, 46, and 48°C, and the optical density was assessed in a Spectronic 20D+ (ThermoSpectronics, Cambridge, United Kingdom).

The proposed minimal standards for describing new taxa of aerobic endospore-forming bacteria (Logan et al., 2009) were followed for the phenotypic characterization of strains 3ASR75-54^T^, 3ASR75-11^T^, and 3ASR75-286. R2A medium supplemented with 7.5% (w/v) salts, and pH adjusted at 7.5 was used for routine growth with an incubation period of 3 days at 37°C. The same methodology was used for reference strains *A. albus* JCM 17364^T^, *A. koreensis* JCM 12387^T^, and *A. salifodinae* JCM 19761^T^. These incubation conditions were used for all the biochemical tests. The protocols for determination of catalase, hydrolysis of gelatin, starch, Tween 80, DNA, casein, and aesculin, production of indole, methyl red and Voges–Proskauer tests, Simmons’ citrate, nitrate and nitrite reduction, H_2_S production, urease, and phenylalanine deaminase are described by Cowan and Steel (1965). A drop of 1% (v/v) tetramethyl-p-phenylenediamine (Kovacs, 1956) was employed to test the oxidase activity in young cultures. Modified phenol red base medium with 0.05% (w/v) yeast extract and supplemented with 7.5% (w/v) salts allowed the determination of acid production. Carbohydrates were filter-sterilized and added to a final concentration of 1% (Cowan and Steel, 1965; Ventosa et al., 1982). In order to test the use of a wide variety of substrates as sole carbon and energy source, or as sole carbon, nitrogen and energy sources, strains were inoculated in the medium described by Koser (1923), as modified by Ventosa et al. (1982). Amino acids, alcohols, and organic acids were supplied to give a final concentration of 1 g L^−1^, and carbohydrates of 2 g L^−1^. All the substrates were added after filter-sterilization.

## 3. Results and discussion

### 3.1. Saline soils sampled from Odiel Saltmarshes Natural Area are heavily contaminated

The heavy metals concentration of the Odiel river waters and sediments have been previously studied due to the past industrial and mining activities in its surroundings, showing high concentrations of arsenic, cadmium, copper, lead, and zinc (Sainz et al., 2002, 2004). The Government of the region of Andalucía, where the area under study is located, sets the following reference criteria for noncontaminated soils (mg kg^−1^): arsenic, 2–5; cadmium, 0.4–0.8; copper, 17–100; lead, 10–50; and zinc, 10–70 (Consejería de Medio Ambiente, 1999). The soils of the Odiel Saltmarshes Natural Area studied here presented values substantially above those ranges (mg kg^−1^): arsenic, 124.3; cadmium, 2.0; copper, 1,853.0; lead, 257.5; and zinc, 443.8, which indicate heavy metal contamination in the sampled soils. This sampling area represents the most contaminated region among the hypersaline soils in the Odiel Saltmarshes Natural Area studied so far (Vera-Gargallo et al., 2019; Galisteo et al., 2023), probably related to its close location to the mouth of the Canal del Burro Grande into the Odiel river. The pH of the sample was 7.04 whereas the electrical conductivity (EC) was 18.49 mS cm^−1^ at 25°C, which is above the 4 mS cm^−1^ at 25°C threshold for defining saline soils (Richards, 1954).

### 3.2. Isolated strains can be split into two groups

**Figure 1:**
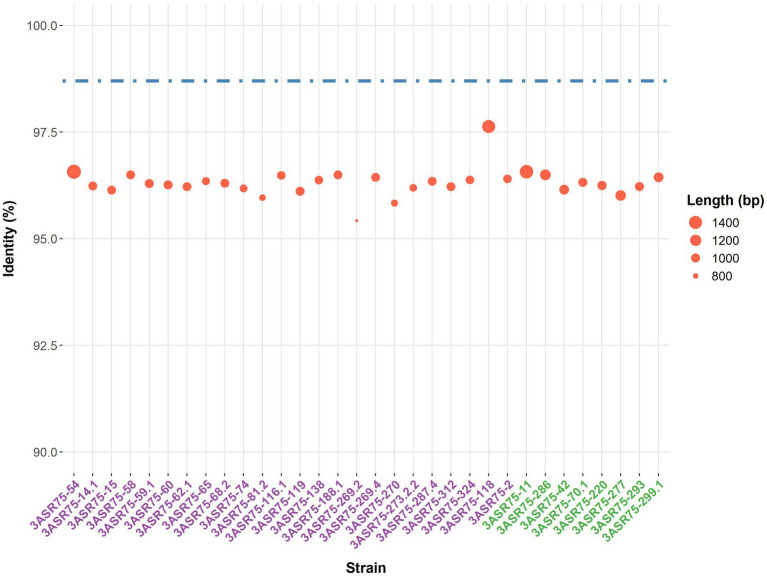
Top hit identity values (%) of the 32 isolates against EzBioCloud database. Best hit was either the species *Aquibacillus koreensis* BH30097^T^ (strain names purple-colored) or *Aquibacillus albus* YIM 93624^T^ (strain names green-colored). Dot size is proportional to the length of the sequenced 16S rRNA gene. Dashed line indicates the 98.7% identity cutoff for species delineation.

**Figure 2:**
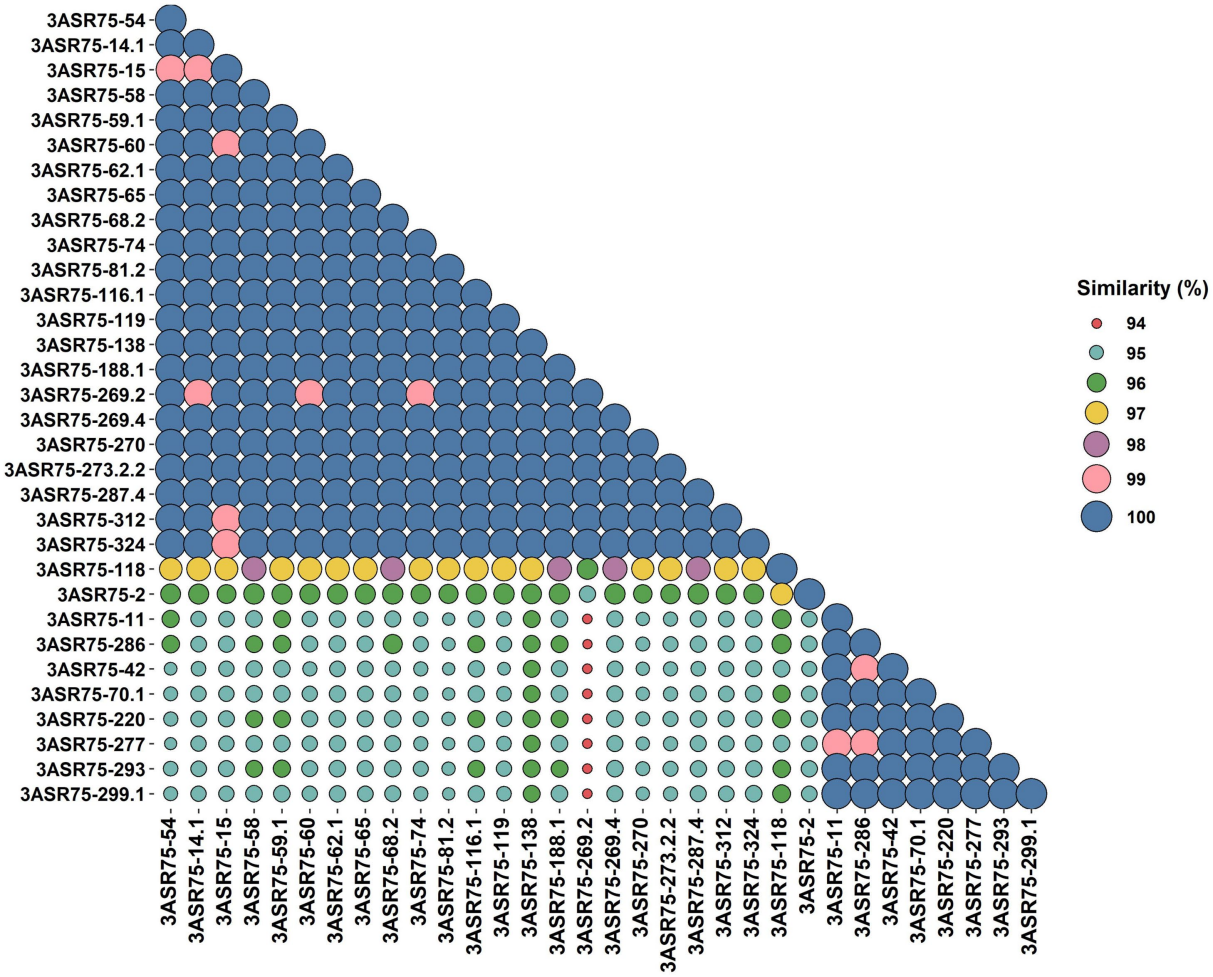
BLASTn identity matrix among the partial or almost complete 16S rRNA gene sequences of the 32 strains isolated in this study.

In a previous study, more than 4,000 strains were isolated in an extensive screening carried out in the hypersaline soils located in the Odiel Saltmarshes Natural Area (Huelva, Southwest Spain) (Galisteo et al., 2023). Out of them, 32 strains showed a close relationship with the genus *Aquibacillus,* according to their partial or almost complete 16S rRNA gene sequence comparison. All the isolates presented a percentage of identity below the 98.65% cutoff for species delineation (Figure 1). Their top hits were either *A. koreensis* BH30097^T^ (97.84–95.51%) for 24 strains or *A. albus* YIM 93624^T^ (97.63–95.51%) for the other eight strains, which seem to indicate that they might be clustered into two groups, denoted as group 1 and group 2. The identity values among themselves exhibited the same pattern for clustering (Figure 2). Within the groups, the identity varied from 100 to 99%, except for the strains 3ASR75-118 and 3ASR75-2, sharing 96.45–96.39% identity with strains of the group 1. In any case, these two strains exhibited higher identity values with respect to members of the group 1 than to members of the group 2, and thus, they both were initially affiliated to the *A. koreensis*-like group 1. Between clusters, the percentage of identity dropped below 97%, indicating that they may constitute two different species. Strains 3ASR75-54^T^ and 3ASR75-11^T^ were selected as type strain of groups 1 and 2, respectively, as they grew well under laboratory conditions and their 16S rRNA genes were sequenced at high quality and long length (1,432 bp and 1,479 bp, respectively).

**Figure 3:**
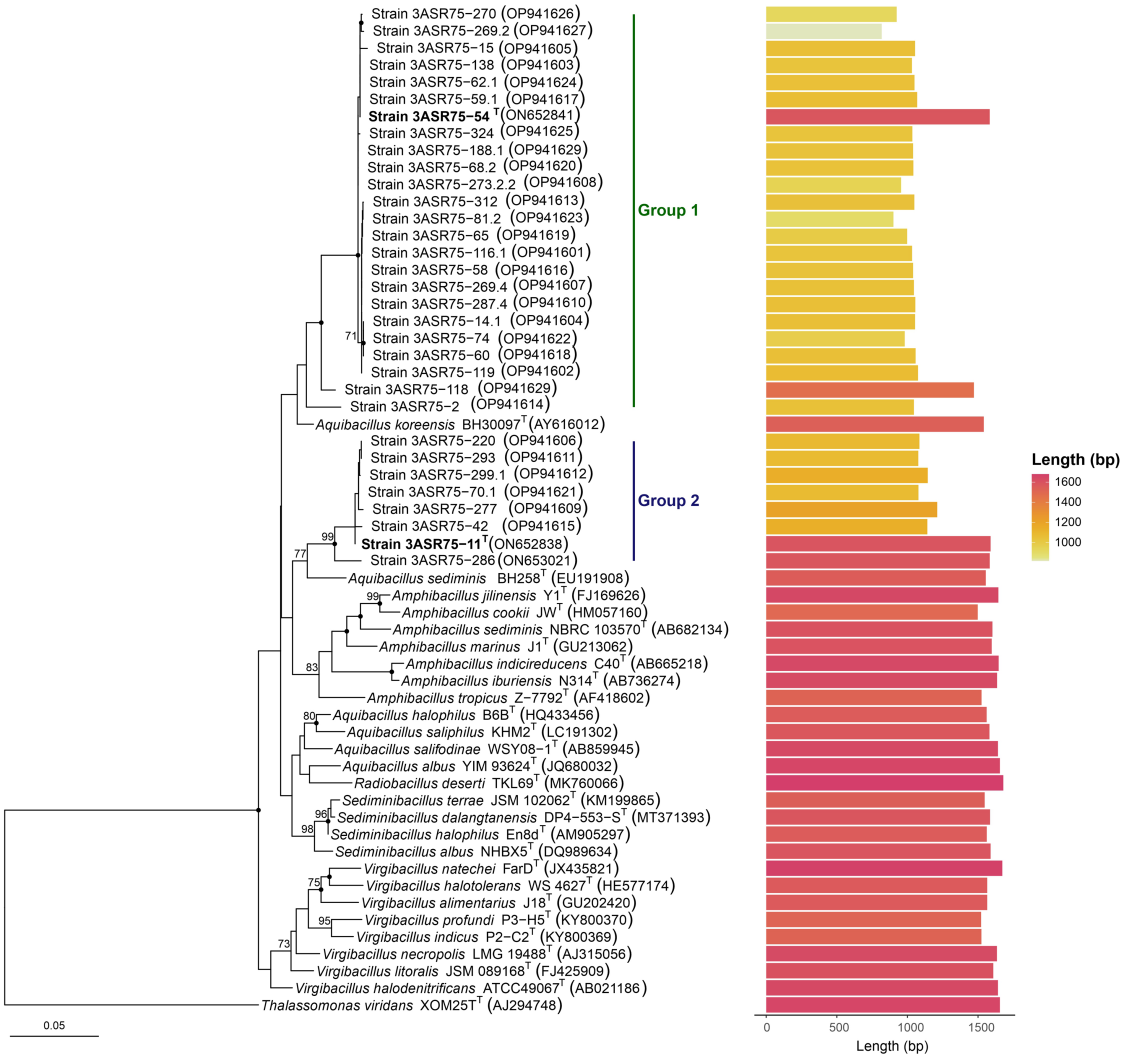
Neighbor-joining phylogenetic tree based on the comparison of the 16S rRNA gene sequences showing the relationships among the new strains and species of the closely related genera *Amphibacillus*, *Radiobacillus*, *Sediminibacillus*, and *Virgibacillus*. Bootstrap values ≥70%, based on 1,000 pseudoreplicates, are indicated above branches. Nodes conserved across the three tree-constructing methods are marked with a filled circle. The species *Thalassomonas viridans* was used as an outgroup. Bar, 0.05 substitutions per nucleotide position.

The phylogenetic tree based on the 16S rRNA gene sequences (Figure 3) provides an enhanced view of the proposed clusters, considering the most discriminative power of phylogenetic methods over identity matrixes. Members of the genus *Aquibacillus* and of the closely related genera *Amphibacillus*, *Radiobacillus*, and *Sediminibacillus,* as well as some representative species of the genus *Virgibacillus*, were included in the phylogenetic analysis. The 24 strains most closely related to *A. koreensis* BH30097^T^ (group 1) clustered together once again, including strains 3ASR75-118 and 3ASR75-2. The eight strains that showed their highest 16S rRNA sequence identity with *A. albus* YIM 93624^T^ also conformed a clear single cluster (group 2). In this latter case, the closest neighbor was not *A. albus* YIM 93624^T^ as expected, but *A. sediminis* BH258^T^. In addition, the branch supporting group 2 displayed a close relationship with species of the genus *Amphibacillus,* turning the genus into polyphyletic. Further incongruences were observed in the tree reconstruction, such as the clustering of the only member of the genus *Radiobacillus, R. deserti,* together with four species of the genus *Aquibacillus,* giving rise to an *Aquibacillus-Radiobacillus* group. Moreover, the species of the genus *Sediminibacillus* formed a monophyletic group neighbor to the *Aquibacillus-Radiobacillus* cluster, constituting a branch separated from other members of the genus *Aquibacillus* and from strains of the groups 1 and 2. Only the genus *Virgibacillus* formed a clearly independent monophyletic branch including all the species within this genus. Clearly, the 16S rRNA gene sequence analysis demonstrated unstable tree topologies since only a few nodes were conserved for all the three tree-constructing algorithms (maximum-likelihood, maximum-parsimony, and neighbor-joining) and bootstrap values were, in general, below 70%. Further analyses considering the whole genome sequences are indispensable to elucidate the correct taxonomic position of the new isolates.

### 3.3. Comparative genomic analyses shed light on the taxonomic status of the new isolates

**Figure 4:**
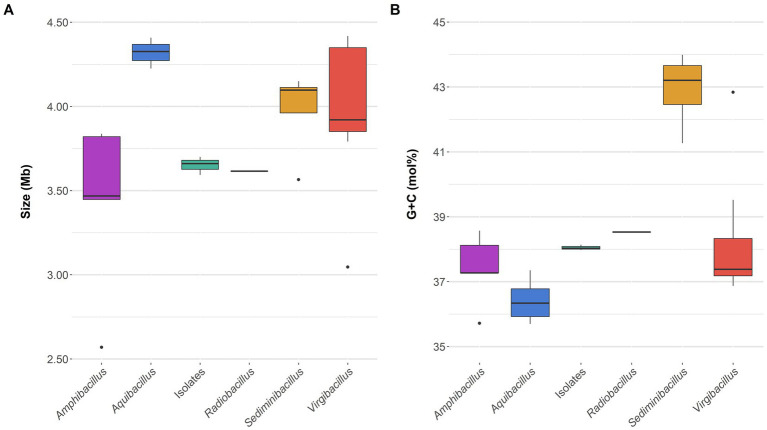
Genome size **(A)** and G + C content **(B)** boxplots of the genome sequences belonging to the family *Bacillaceae* included into this study. The three new isolates exhibited a very similar value among them and with respect to the single species of the genus *Radiobacillus*.

The draft genome sequence of the selected type strains 3ASR75-54^T^ (GCF_028416595.1) and 3ASR75-11^T^ (GCF_028416575.1) were *de novo* obtained. Moreover, we sequenced an additional reference strain from group 2 (i.e., strain 3ASR75-286, GCF_028416555.1), given the placement of this group next to the genus *Amphibacillus*, which made us suspicious of group 2 forming a new separated genus. The genomes of the mentioned strains were assembled in 70, 71, and 67 contigs, respectively. Their total genome size and G + C content ranged 3.59–3.70 Mb and 38.0–38.1 mol%, although the first parameter was slightly higher for strain 3ASR75-54^T^ (Supplementary Table S2). Comparisons with closely related genera showed that genome size (Figure 4A) and G + C content (Figure 4B) of the new strains were more similar to those of the genus *Radiobacillus*. On the contrary, the species of the genus *Aquibacillus* possessed larger genomes, 4.22–4.41 Mb, and a lower G + C content, 35.7–37.4 mol%, which reinforces the idea of the new isolates not belonging to the previously described species of this genus (Supplementary Table S2; Figures 4A,B). The genome of strain 3ASR75-54^T^ encoded 3,535 CDS, 100 tRNA, and 15 rRNA, whereas the genomes of strains 3ASR75-11^T^ and 3ASR75-286 encoded 3,590 and 3,617 CDS, respectively, and harbored less RNA sequences than strain 3ASR75-54^T^ (66 tRNA and 9 rRNA) (Supplementary Table S2). The DNA of the reference species *A. koreeensis* JCM 12387^T^ was likewise sequenced (GCF_028416535.1) as it was not available at the beginning of this study. This genome sequence was assembled into 56 contigs with a total size of 4.33 Mb and a G + C content of 36.8 mol% (Supplementary Table S2), in agreement with the other six genomes of type strains available for the genus *Aquibacillus*. Further genomic features are shown in Supplementary Table S2.

**Figure 5:**
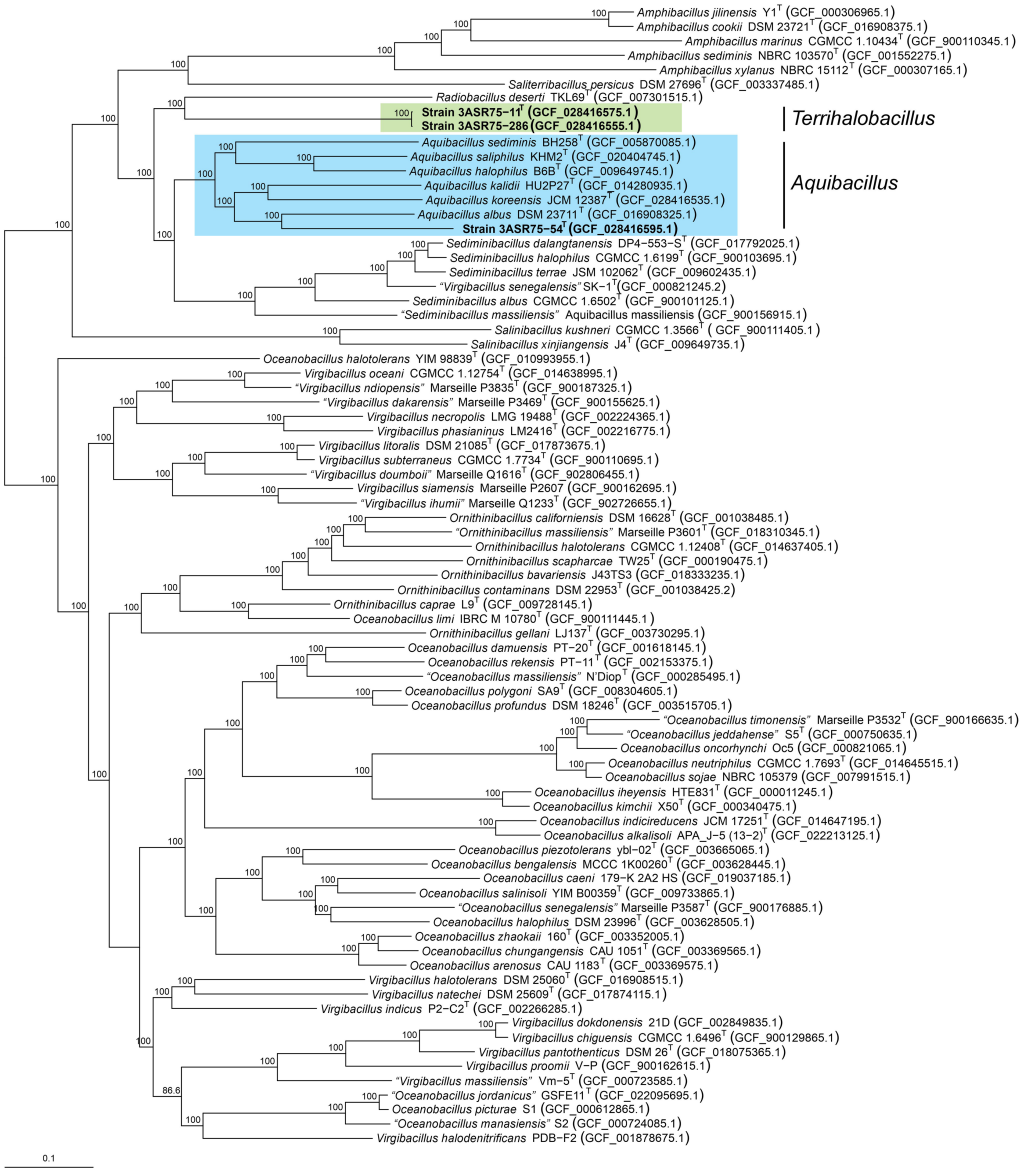
Approximately maximum-likelihood phylogenomic tree based on 739 concatenated core protein sequences showing the relationships between the novel isolates and 79 closely related species of the family *Bacillaceae*. Bootstrap values ≥70% are indicated above the respective branch. Bar, 0.1 substitutions per nucleotide position.

The genus *Aquibacillus* belongs to a large family, *Bacillaceae*, along with other 116 genera with validly published names (Parte et al., 2020; last consulted on 10/02/2023). Considering the weak robustness of the 16S rRNA gene-based phylogenetic tree as stated above, a more reliable phylogenomic tree was constructed based on a large set of genomes from the genus *Aquibacillus* and neighbor genera. The 739 protein-based approximately maximum-likelihood inference included a total of 76 species from nine genera, as well as the three new strains and *A. koreensis* JCM 12387^T^, whose genomes were sequenced in this study (Figure 5). Unlike the single 16S rRNA gene phylogeny (Figure 3), most branches were now supported by a 100% bootstrap value, providing a consistent topology to elucidate the evolutionary relationship between the new isolates and the closely related taxa. All the described species of the genus *Aquibacillus* clustered together in a monophyletic group, including the recently sequenced genomes of *A. koreensis* JCM 12387^T^ and the new strain 3ASR75-54^T^. Phylogenomic analysis displayed the closest relationship of strain 3ASR75-54^T^ with *A. albus*, even if its 16S rRNA gene sequence showed a higher percentage of identity with *A. koreensis* (Figures 1, 3). On the other hand, strains 3ASR75-11^T^ and 3ASR75-286, whose BLASTn top hit was *A. albus* (Figure 1) and the closest neighbor according to the 16S rRNA gene sequence phylogeny was *A. sediminis* (Figure 3), now constitute a single branch related to the single species of the genus *Radiobacillus*. A first glimpse might affiliate strains 3ASR75-11^T^ and 3ASR75-286 with a novel species of the genus *Radiobacillus*. However, the length of branch connecting both strains to the node shared with *R. deserti* is sufficiently large to consider strains 3ASR75-11^T^ and 3ASR75-286 as members of a different genus. Further analyses are needed in order to confirm this hypothesis.

**Figure 6:**
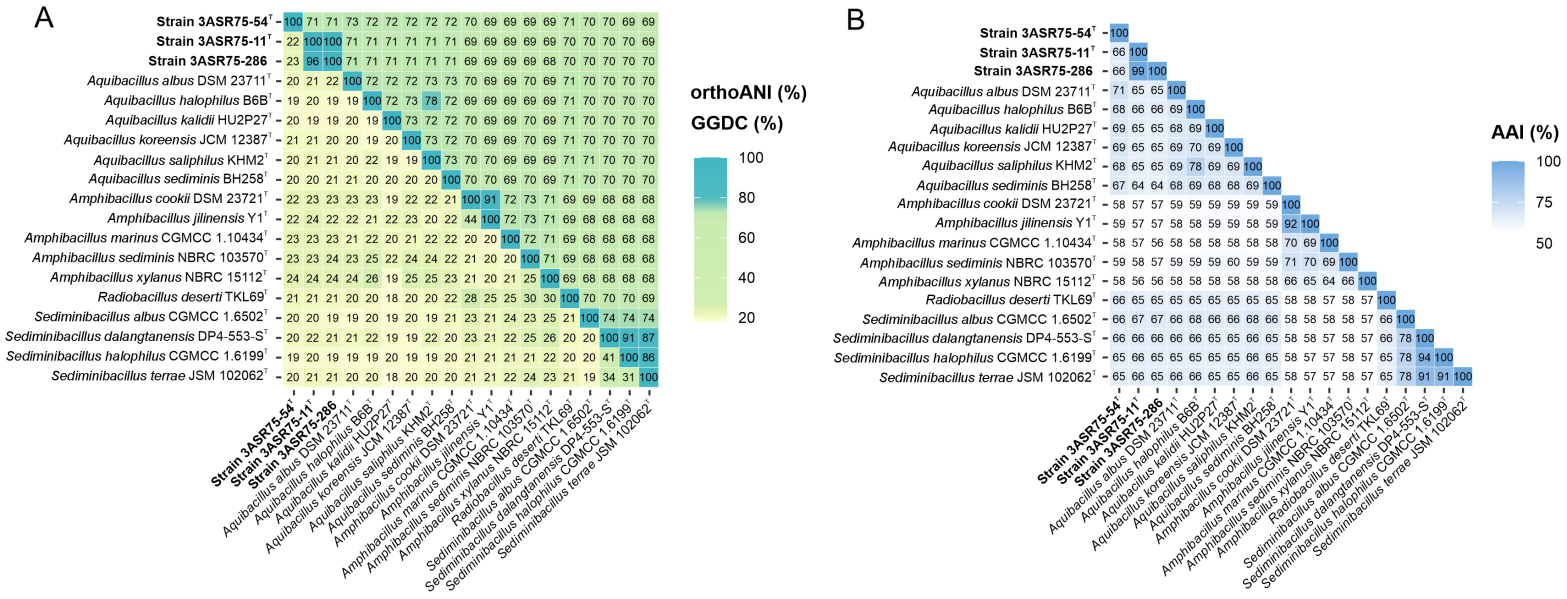
OrthoANI (upper triangle)/GGDC (lower triangle) **(A)** and AAI **(B)** values (%) among strains 3ASR75-54^T^, 3ASR75-11^T^, 3ASR75-286, and the closest related members of the genera *Aquibacillus*, *Radiobacillus*, and *Sediminibacillus*. OrthoANI/GGDC results confirmed that strains 3ASR75-54^T^, 3ASR75-11^T^, and 3ASR75-286 cannot be affiliated to any of the currently described species of these genera and also proved that strains 3ASR75-11^T^ and 3ASR75-286 constituted a single species. AAI data unequivocally assigned strain 3ASR75-54^T^ to the genus *Aquibacillus* and suggested the placement of strains 3ASR75-11^T^ and 3ASR75-286 into a new genus within the family *Bacillaceae*.

Concerning OGRIs analyses, dDDH and orthoANI values were estimated between the three new isolates and the species of the genera *Aquibacillus*, *Amphibacillus*, *Radiobacillus*, and *Sediminibacillus*, which are the closest related genera within the family *Bacillaceae* (Figure 6A). The highest dDDH percentage obtained was 24.2%, which is far below the 70% cutoff for species delineation (Stackebrandt and Goebel, 1994; Auch et al., 2010). Similarly, orthoANI results were all equal or lower than 72%, again lower that the 95% threshold established for species differentiation (Goris et al., 2007; Richter and Rosselló-Móra, 2009; Chun and Rainey, 2014). Nevertheless, the outcome between strains 3ASR75-11^T^ and 3ASR75-286 exceeded both limits, with values of 96.5 and 100% for dDDH and orthoANI, respectively. Thus, we can conclude that our isolates constitute two novel species, one represented by strain 3ASR75-54^T^ and the other comprising strains 3ASR75-11^T^ and 3ASR75-286.

**Figure 7:**
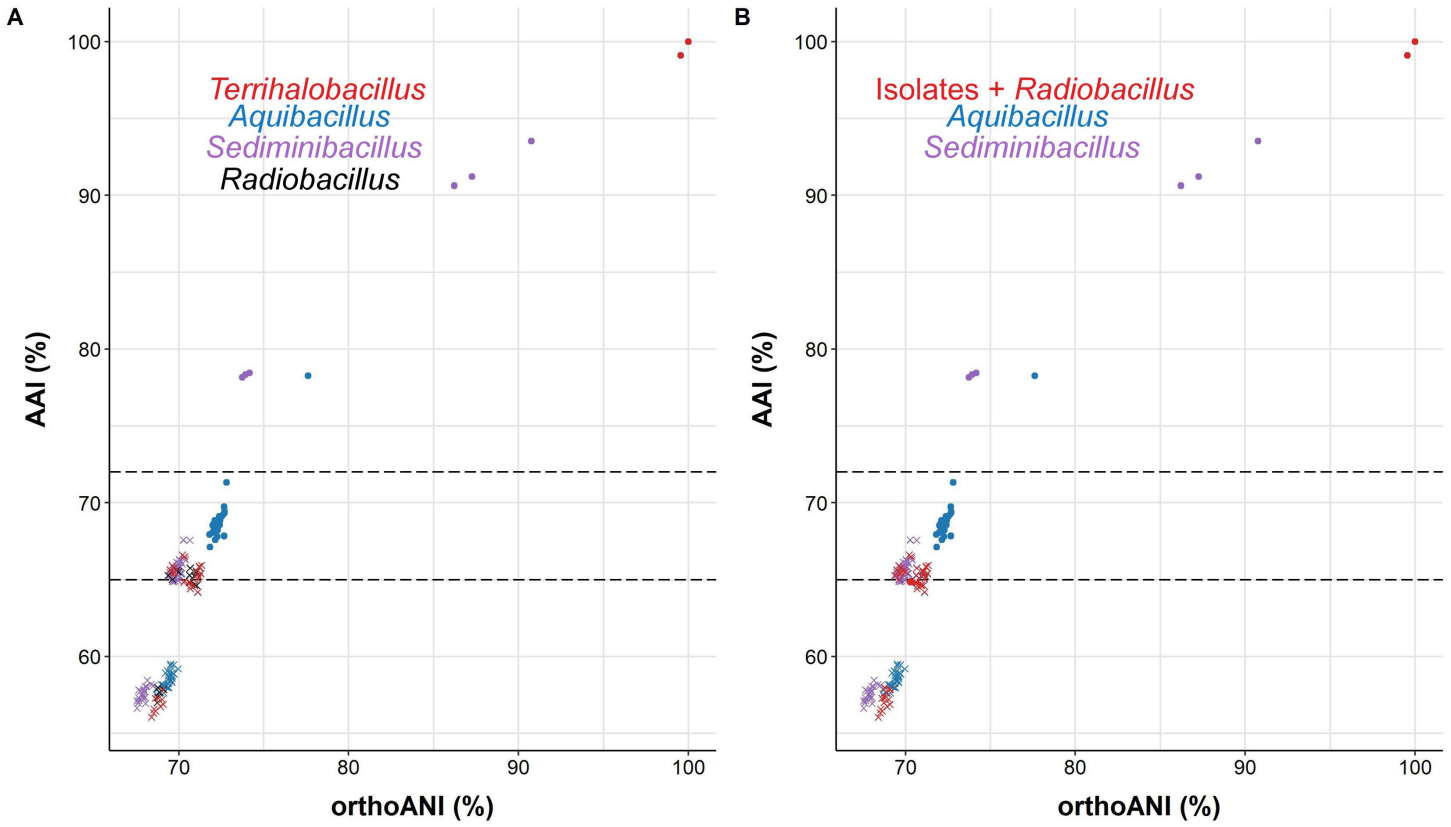
Scatter plot displaying AAI-orthoANI pairs of values for the studied genomes within and between genera assuming the placement of strains 3ASR75-11^T^ and 3ASR75-286 into the new genus *Terrihalobacillus* **(A)** and into the genus *Radiobacillus* **(B)**. Dotted lines denote the 65–72% AAI cutoff for genus delineation. Strain 3ASR75-54^T^ has been included among the species of the genus *Aquibacillus*. Circles and crosses indicate intra-and inter-genus values, respectively.

Another widely used OGRI is AAI, which considers that pairs of genomes with values lower than 72–65% belong to species of different genera (Konstantinidis and Tiedje, 2007; Konstantinidis et al., 2017). In particular, we can observe that species within the genus *Aquibacillus* shared AAI percentages between 67.6 and 69.7% (Figure 6B). The new species represented by strain 3ASR75-54^T^ exhibited an AAI range of 71.3-67.1% with species of *Aquibacillus*, while the values were fairly distant with other closely related genera (the highest being 66.2% with *Sediminibacillus albus*). These data, along with the robust topology of the phylogenomic tree, clearly indicate that the strain 3ASR75-54^T^ belongs to a non-yet described species of the genus *Aquibacillus*. On the other hand, the AAI values of strains 3ASR75-11^T^ and 3ASR75-286 with respect to the species of the genus *Aquibacillus* varied between 65.9–64.2%, lower than the current intrageneric range for *Aquibacillus* and in the lower bound or below the accepted 72–65% cutoff for genus delineation. The AAI comparisons between strains 3ASR75-11^T^ and 3ASR75-286 and the other closely related genera within the *Bacillaceae* showed the highest values for the genus *Sediminibacillus* (66.6–65.8%), which might suggest their affiliation to this genus. However, the phylogenomic tree (Figure 5) allows us to discard this thesis considering their polyphyly. As stated before, genomic-based inference groups strains 3ASR75-11^T^ and 3ASR75-286 with *R. deserti*, supporting their placement into the genus *Radiobacillus*. This assumption is not well supported because AAI values between the two taxa (64.9–64.8%) contravenes the AAI threshold for genus delineation. Nevertheless, it must be noted that only one species of the genus *Radiobacillus* is described to date, turning impossible to predict if future descriptions of *Radiobacillus* species will entail the rise in the upper bound AAI range, making feasible the grouping of strains 3ASR75-11^T^ and 3ASR75-286 as members of the genus *Radiobacillus*. Therefore, we do not have a sound scientific evidence to assert whether those two strains are part of the genus *Radiobacillus* or, on the contrary, they constitute a novel genus within the family *Bacillaceae*. In order to elucidate this issue, we plotted the AAI-orthoANI pairs of values for the studied genomes within and between genera (Figure 7). When strains 3ASR75-11^T^ and 3ASR75-286 were considered as a separated genus, the inter-and intra-genus results do not overlap (Figure 7A). However, the clustering of strains 3ASR75-11^T^ and 3ASR75-286 with the genus *Radiobacillus* gave rise to an intra-genus spot located within the inter-genus point cloud (Figure 7B). Consequently, our results suggest that the species constituted by strains 3ASR75-11^T^ and 3ASR75-286 does not belong to any of the currently described genera within the *Bacillaceae* and should be accommodated in a novel genus.

**Figure 8:**
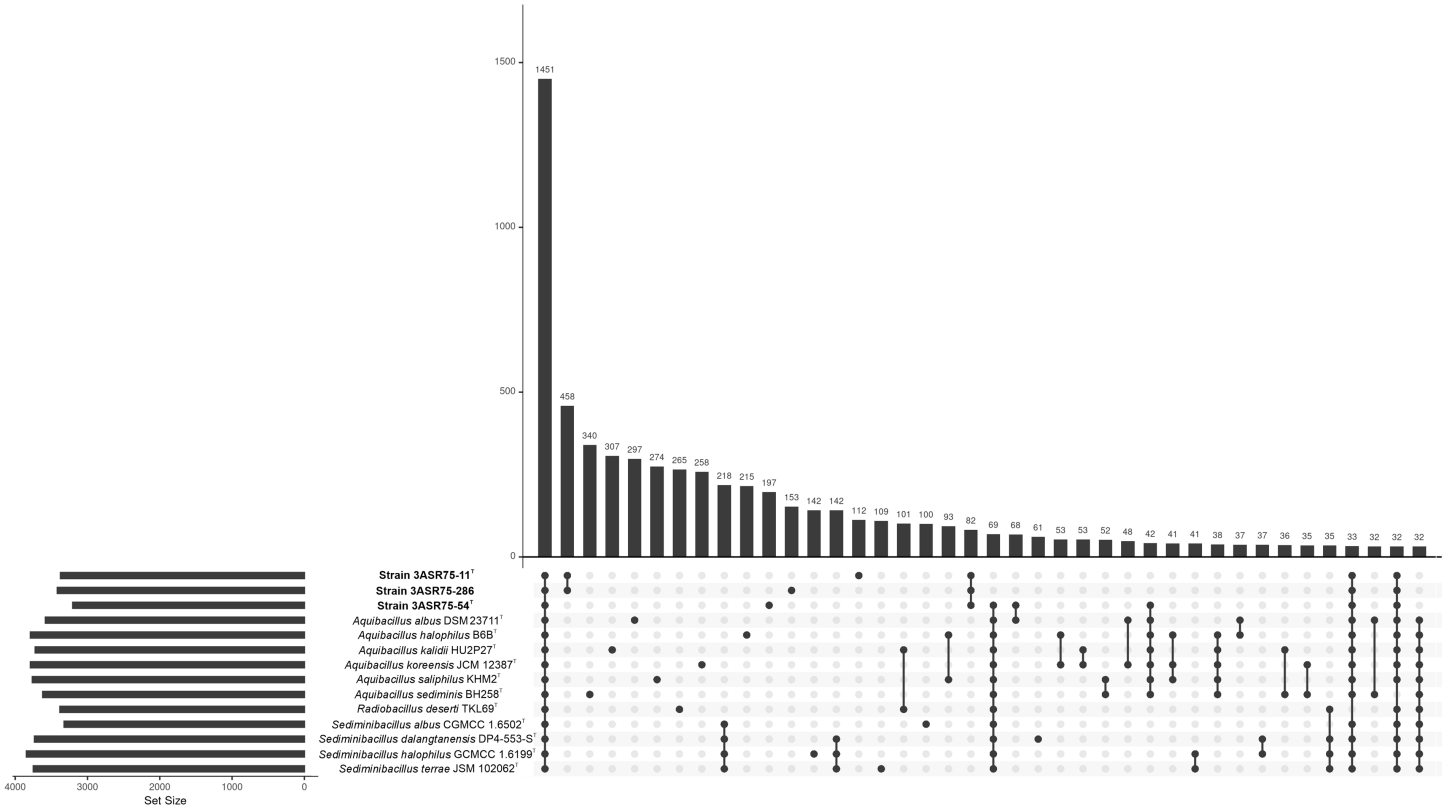
Upset plot showing the intersecting orthologous genes of the isolated strains 3ASR75-54^T^, 3ASR75-11^T^, and 3ASR75-286, and the species of the closely related genera *Aquibacillus*, *Radiobacillus*, and *Sediminibacillus*. The core genome encoded a total of 1,451 genes out of a pangenome of 18,524 genes. Isolated strains 3ASR75-54^T^, 3ASR75-11^T^, and 3ASR75-286 harbored an accessory genome with 197, 112, and 153 exclusive genes, respectively. A total of 458 genes were solely present in strains 3ASR75-11^T^ and 3ASR75-286, while 82 genes where exclusively shared between the three isolates.

The genomes of strains 3ASR75-54^T^, 3ASR75-11^T^, and 3ASR75-286, together to those of the type strains of the species of the genera *Aquibacillus*, *Radiobacillus*, and *Sediminibacillus* shared a total of 1,451 core genes. Part of the accessory genome is also common for some of the studied species; however, a great number of strain-specific genes were detected. Excluding the core genome, the larger cluster of genes (458) was that shared by the isolated strains 3ASR75-11^T^ and 3ASR75-286. The former harbored 153 strain-exclusive genes while the latter 112. Out of our three isolates, strain 3ASR75-54^T^ was the one containing the higher number of singletons (197). Furthermore, those three strains shared more orthologous genes between them (82) than with any of the other genomes under study. Remarkably, strains 3ASR75-11^T^ and 3ASR75-286 did not display a significant number of common genes with any particular genus, even with their closest relative, the genus *Radiobacillus* (Figure 8). A further insight about the functions encoded in the accessory genome is described in section 3.5. Besides, the observed variability between species and between strains of the same species may indicate their specialization either for adaptation to a specific habitat or to carry out an ecological role in the community.

### 3.4. Chemotaxonomic and phenotypic analyses support the new taxa descriptions as members of the family *Bacillaceae*

Strain 3ASR75-54^T^ exhibited a chemotaxonomic profile similar to that of the other members of the genus *Aquibacillus* (Supplementary Table S3), with anteiso-C_15:0_ as the most abundant fatty acid (47.9%), followed by iso-C_15:0_ (11.6%), and anteiso-C_17:0_ (10.7%). Strain 3ASR75-11^T^ showed a high predominance of anteiso-C_15:0_ (66.4%), whereas other minor fatty acids present were anteiso-C_17:0_ (9.9%) and iso-C_16:0_ (6.8%). This fatty acid composition of strain 3ASR75-11^T^ was comparable to that of the species of the closely related genera *Aquibacillus*, *Radiobacillus*, and *Sediminibacillus* (Lee et al., 2006; Carrasco et al., 2008; Zhang et al., 2012, 2015; Amoozegar et al., 2014; Lee and Whang, 2019; Li et al., 2020; Wang et al., 2021; Cho and Whang, 2022).

Major morphological and physiological characteristics of the new strains (Table 1) were consistent with those described for members of the family *Bacillaceae*. Cells were rod-shaped, endospore-forming, and motile. Colonies were circular and white pigmented. Strain 3ASR75-54^T^ displayed an optimal NaCl concentration supporting growth similar to other members of the genus *Aquibacillus*. On the other hand, strain 3ASR75-11^T^ grew optimally at lower NaCl concentrations than species of the genera *Aquibacillus* and *Radiobacillus*. Further biochemical characteristics are detailed in the new species descriptions and in Supplementary Table S4. The phenotypic similarities found between the strains 3ASR75-54^T^, 3ASR75-11^T^, and 3ASR75-286 and the studied members of the family *Bacillaceae* reinforce the proposal for their placement within this family. At the same time, features such as motility and optimal NaCl concentration for growth allow their differentiation from the closely related species.

**Table 1:** Differential features of the new isolated strains and closely related species.

Characteristics	1	2	3	4^a^	5^b^	6^c^	7^d^	8^e^	9^f^	10^g^	11^h^
Cell size	0.7–1.0 × 2.8-3.0	0.7–1.0 × 3.0–5.0	0.5–0.7 × 3.0–5.0	0.3–0.5 × 2.0–6.0	0.5–0.7 × 2.0–4.0	0.5–0.7 × 2.0–4.0	0.3–0.5 × 4.0–6.0	0.4–0.6 × 5.0–8.1	0.7–0.8 × 1.2–2.9	0.7–1.2 × 3.5–5.0	0.2–0.6 × 1.4–5.6
Colony pigmentation	White	White	White	White	White	Milk	White	Cream	White	Cream	White
Motility	+	+	+	NA	+	+	+	+	+	+	−
NaCl range (%, w/v)	0.5–17	0.5–20	NA	1–17	0–14	0–14	0.5–20	0–10	1–20	0.5–20	0–12
NaCl optimum (%, w/v)	7	2	NA	5–10	5–8	5–8	10	4	10	7–10	0–8
pH range	6–8	4–9	NA	4–9	5–12	6–9	6.5–9	6–9	6–10	5.5–9	6–9
pH optimum	6	5	NA	7	7	7	7	7	8	7	6–8.5
Temperature range (° C)	11–45	10–45	NA	15–45	10–40	10–40	20–40	25–45	10–45	15–45	20–50
Temperature optimum (° C)	37	37	NA	25–30	30	30	35	37	37	35	35
Anaerobic growth	+	+	+	−	−	−	−	−	+	NA	−
Aesculin hydrolysis	+	−	−	+*	+	+	+*	+*	+	+	NA
Urease	−	−	−	+*	−	+	+*	+*	NA	+	−
Nitrite reduction	+	−	−	+*	−	+	−*	−*	NA	+	NA
G + C content (mol%, genome)	38.0	38.0	38.1	36.7	35.9	36.0	36.8	36.9**	35.7	37.4	38.5

1. Strain 3ASR75-54^T^; 2. Strain 3ASR75-11^T^; 3. Strain 3ASR75-286; 4. *Aquibacillus albus* YIM 93624^T^ (G + C content from *A. albus* DSM 23711^T^); 5. *Aquibacillus halophilus* B6B^T^; 6. *Aquibacillus kalidii* HU2P27^T^; 7. *Aquibacillus koreensis* BH30097^T^ (G + C content from *A. koreensis* JCM 12387^T^); 8. *Aquibacillus salifodinae* WSY08-1^T^; 9. *Aquibacillus saliphilus* KHM2^T^; 10. *Aquibacillus sediminis* BH258^T^; 11. *Radiobacillus deserti* TKL69^T^. NA, not available. *, data from this study. **, determined by HPLC.

a Zhang et al. (2012);

b Amoozegar et al. (2014);

c Wang et al. (2021);

d Lee et al. (2006);

e Zhang et al. (2015);

f Cho and Whang (2022);

g Lee and Whang (2019);

h Li et al. (2020).

### 3.5. Functional genomic analysis correlates with the phenotype and reveals the existence of a variety of cell membrane transporters

A total of 1,584 KO were identified for strain 3ASR75-54^T^. Out of them, 21 were exclusive of this strain, that is, not present in the genome of any of the studied species of the genera *Aquibacillus*, *Radiobacillus*, and *Sediminibacillus*, nor in that of the strains 3ASR75-11^T^ and 3ASR75-286. Among these functions, strain 3ASR75-54^T^ exhibited a putative zinc/manganese transport system (K02074, K02075, and K02077); the large (*nirB* gene) and small (*nirD* gene) subunits of the nitrite reductase enzyme turning nitrite into ammonia (K00362 and K00363); and CRISPR-associated protein-coding genes, such as *cas3*, *cas4*, *csd1*, *csd2*, and *cas5d* (K07012, K07464, K19117, K19118, and K19119).

The genomes of strains 3ASR75-11^T^ and 3ASR75-286 were annotated with 1,604 and 1,573 KO, respectively. Strain 3ASR75-11^T^ possessed 61 KO that were not present in strain 3ASR75-286, while 3ASR75-286 only presented 30 KO not detected in strain 3ASR75-11^T^. Those 61 strain-specific functions encoded mostly saccharide transporters (multisugar, ribose/autoinducer 2/D-xylose, and rhamnose), as well as the ability to transform sorbitol into sorbitol 6-phosphate (K02781, K02782, and K02783) in strain 3ASR75-11^T^. On the other hand, strain 3ASR75-286 harbored singular metal related functions, such as copper efflux regulator (K19591), cadmium/lead responsive transcriptional repressor (K21885), and iron-siderophore transporter system permease protein (K25111). Besides their differences, both strains displayed 35 common functions that were not present in any other of the studied genomes of the closest related genera. Among them, we could identify transporters, such as those for arginine/lysine/histidine (*artPQM* genes; K17077, K23059, and K23060), chitobiose (c*hiEFG* operon; K17244, K17245, and K17246), and zinc and cadmium (ZIPB; K16267), which passively uptakes these ions into the cytoplasm (Lin et al., 2010); and Ca^2+^/H^+^ antiporter (*chaA*; K07300), working as a K^+^ extrusion system to maintain K^+^ homeostasis under salt stress conditions (Radchenko et al., 2006). Enzyme-coding genes were also identified among the 35 shared KO, such as those for N-methylhydrantoinase (*hyuAB*; K01473 and K01474), glutaconate CoA-transferase subunits A and B (*gctAB*; K01039 and K01040), and ferritin (K02217). Additionally, the genome of strain 3ASR75-11^T^ was annotated with 10 KO not present in any of the other analyzed genomes, although these functions do not seem to provide any relevant feature. On the other hand, strain 3ASR75-286 harbored two KO related to heavy metal resistance among its 10 exclusive KEGG Orthology identifiers, one being the abovementioned copper efflux regulator (K19591) and the second an alkylmercury lyase (K00221). Further heavy metal tolerance mechanisms are discussed in section 3.8.

All the three strains under study, 3ASR75-54^T^, 3ASR75-11^T^, and 3ASR75-286, contained between 39 and 41 KO related to the sporulation process, like the species of the genera *Aquibacillus*, *Radiobacillus*, and *Sediminibacillus* (Supplementary Figure S1). This in-silico analysis agrees with the morphological observation revealing the formation of terminal endospores at the poles of cells after incubation at 37°C for 7 days. Additionally, the three strains harbored the response regulator for oxygen limitation (K07651, K07775, and K02259), which correlates with their anaerobic growth in laboratory conditions (Table 1). Moreover, although the information encoded into their genome sequences suggests the existence of mechanisms for low-temperature tolerance, the strains 3ASR75-54^T^, 3ASR75-11^T^, and 3ASR75-286, as well as the other closely related species of the genera *Aquibacillus* and *Radiobacillus* have only shown growth above 10°C. On the contrary, the high-temperature tolerance deducted from the genome sequence has also been tested *in vitro*, with the bacteria being able to survive up to 50°C.

### 3.6. A putatively functional molybdenum cofactor biosynthetic pathway identified in the new isolates and species of the genus *Aquibacillus*

Since the closest described evolutionary relative of strains 3ASR75-11^T^ and 3ASR75-286 was the sole species of the genus *Radiobacillus* (Figure 5), an in-depth comparison of their genome-inferred metabolisms was explored in order to unveil their main similarities and differences. BlastKOALA annotation yielded 1,601 KO for the genome sequence of *R. deserti*, of which 284 were absent in the genomes of strains 3ASR75-11^T^ and 3ASR75-286, whereas 248 KO identified for the new isolated strains were missing in *R. deserti*. The single species of the genus *Radiobacillus* harbored metal transporters, such as those for iron (III) (K02010, K02011, and K02012), iron-siderophore (K23185, K23186, K23187, and K23188), manganese (K19975, K19976, and K19973), and zinc (K09815, K09816, and K09817). Besides, we found a mechanism for acid tolerance and Na^+^ transporters encoded in the genome of *R. deserti,* but not in those of strains 3ASR75-11^T^ and 3ASR75-286. Conversely, strains 3ASR75-11^T^ and 3ASR75-286 possessed heme transporters (K02193, K02194, and K02195), as well as iron (III) citrate transport systems (K23181, K23182, K23183, and K23184). More significantly, strains 3ASR75-11^T^ and 3ASR75-286 presented ABC-type molybdate transporters (*modABC* genes; K02020, K02018, and K02017) and the biosynthetic pathway for the molybdenum cofactor (Moco), a relevant molecule in all domains of life (Hille, 1996; Leimkühler, 2020). Molybdoenzymes, or enzymes in which the active metal is molybdenum (Mo), are widespread in prokaryotes and eukaryotes, and more than 60 different molecules have been described to date (Hille et al., 2014; Peng et al., 2018). Organisms encoding them also harbors Moco biosynthetic and transport pathways in their genome (Peng et al., 2018). Those enzymes are mostly involved in redox reactions (Hille, 1996), with a key role in the metabolism of nitrogen, sulfur, and carbon compounds (Schoepp-Cothenet et al., 2012; Hille et al., 2014; Magalon and Mendel, 2015), and are related to anaerobic respiration in bacteria (Zupok et al., 2019). According to their Mo centers, they are divided into the xanthine oxidase (XO) family, the sulfite oxidase (SO) family, and the DMSO reductase family (Hille, 1996).

**Figure 9:**
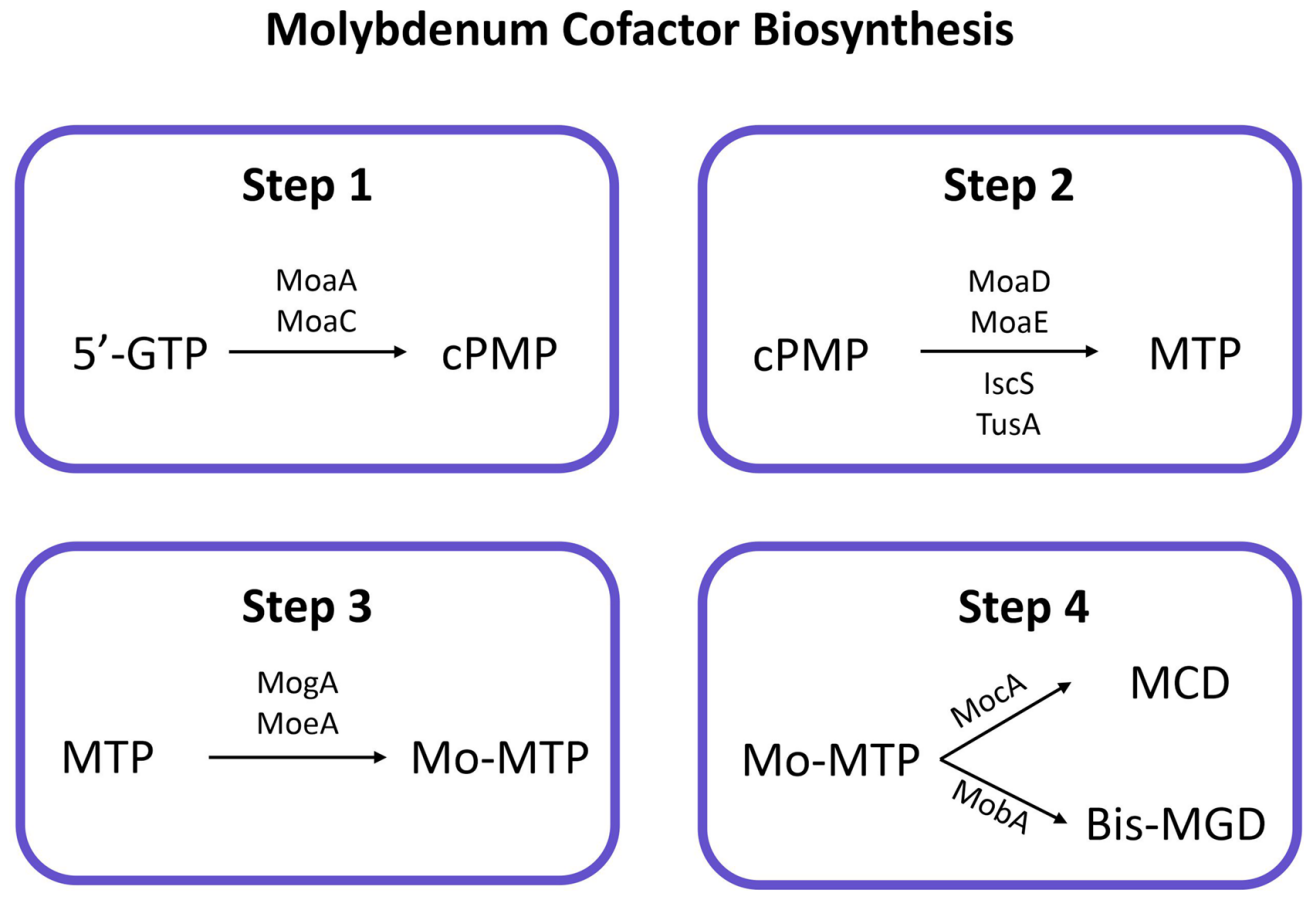
Conserved biosynthesis pathway for molybdenum cofactor in prokaryotes. Enzymes catalyzing each reaction are indicated next to the arrow. 5′-GTP, 5′-guanosine triphosphate; cPMP, cyclic pyranopterin monophosphate; MPT, molybdopterin; Mo-MTP, molybdenum inserted in MTP; MCD, MPT cytosine dinucleotide; bis-MGD, MPT guanosine dinucleotide.

Several genes organized into five operons (*moaABCDE*, *mobAB*, *mocA*, *moeAB*, and *mogA*) (Shanmugam et al., 1992) had been related to the biosynthesis of Moco and one operon (*modABCD*) to the molybdate uptake system (Walkenhorst et al., 1995). Most of them were identified in the genomes of strains 3ASR75-54^T^, 3ASR75-11^T^, 3ASR75-286, and all the species of the genus *Aquibacillus*, but not in those of the genera *Sediminibacillus* and *Radiobacillus* (Supplementary Table S5). Moco biosynthetic pathway is highly conserved and there is evidence that it was once encoded by the last universal common ancestor (Schoepp-Cothenet et al., 2012; Magalon and Mendel, 2015). Though complex, Moco biosynthesis is well understood in prokaryotes (Zupok et al., 2019; Leimkühler, 2020) and it can be divided into four steps: (a) 5′-GTP (5′-guanosine triphosphate) is transformed into cPMP (cyclic pyranopterin monophosphate) by MoaA [a two (4Fe-4S)-cluster-containing enzyme] and MoaC (Wuebbens and Rajagopalan, 1993); (b) two sulfur atoms are inserted into cPMP by MoaD and MoaE (Pitterle et al., 1993), with IscS and TusA proteins involved in the sulfur-transfer process (Leimkühler, 2020), leading to molybdopterin (MPT); (c) MogA and MoeA add Mo to the molecule obtaining Mo-MTP (=Moco) (Joshi et al., 1996); (d) Mo-MTP can now be used as a cofactor by proteins of the SO family (Brokx et al., 2005; Havelius et al., 2011) or can be further modified to MCD (MPT cytosine dinucleotide) or bis-MGD (MPT guanosine dinucleotide) by MocA and MobA, respectively (Neumann et al., 2011; Reschke et al., 2013) (Figure 9). None of the studied genomes encoded the *mogA* gene, involved in step 3 of Moco biosynthesis. Nevertheless, when the surrounding medium possesses a high concentration of molybdate (>1 mM), the reaction catalyzed by the ATP-dependent MogA has been demonstrated not to be essential (Neumann and Leimkühler, 2008). Furthermore, the *moeB* gene, whose function is not-yet known, was only detected in strain 3ASR75-54^T^ and some species of *Aquibacillus*. However, considering that this study was based on draft (not complete) genomes (Supplementary Table S2) and that Moco biosynthetic pathway is highly conserved in organisms with Mo-dependent enzymes (Zhang et al., 2011; Hille et al., 2014), we could safely assume that the new isolates and all the species of the genus *Aquibacillus* possess a putatively functional biosynthetic route for this cofactor. No molybdoenzymes were found in the proteomes of species of *Radiobacillus* and *Sediminibacillus*, in agreement with the lack of Moco biosynthetic pathway, but some Mo-requiring enzymes were encoded by strains 3ASR75-54^T^, 3ASR75-11^T^, and 3ASR75-286, especially from the DMSO reductase family, the most prevalent family of molybdoenzymes in both bacteria and archaea (Zhang et al., 2011). Among them, we could highlight the presence of nitrate reductase *narGHI* (K00370, K00371, K00374) in strains 3ASR75-11^T^, 3ASR75-286, *A. kalidii* and *A. sediminis*, and arsenite oxidase *aoxA* (K08355) in strain 3ASR75-54^T^ and the studied species of *Aquibacillus,* except for *A. sediminis*, in both cases with activities in the respiratory chain (Hille et al., 2014; Miralles-Robledillo et al., 2019).

The biosynthesis of Moco is positively regulated by ModE (Anderson et al., 2000), although this protein was not encoded in any of the studied genomes. However, the carbon storage regulator CsrA has been proved to enhance the Moco synthesis under conditions of high demand (Zupok et al., 2019), and its coding gene was present in all the analyzed genomes, including those from the genera *Sediminibacillus* and *Radiobacillus*.

The functional annotation of the metagenomic dataset SMO1 showed the presence of KO identifiers involved in the Moco biosynthesis among the 317,837 total assigned KO numbers. However, only 4 to 6 copies have been annotated for the proteins constituting the ModABC molybdate transporter. Therefore, the uptake of Mo from the media is not a widespread feature among the prokaryotic inhabitants of the hypersaline soils from the Odiel Saltmarshes Natural Area. The presence of this transporter in the genome of our isolates may indicate that their metabolisms highly rely on molybdenum related proteins and ensure the Mo uptake by specific transporters, whereas other molybdenum-requiring microorganisms acquiree Mo using other more unspecific mechanisms. Besides, molybdoenzyme *aoxA*, present in the genomes of 3ASR75-54^T^ and most members of the genus *Aquibacillus*, was not found in the SMO1 proteome.

To sum up, Moco biosynthesis is a well conserved pathway in prokaryotes. Its absence usually involves the lack of Mo-dependent proteins. Our genome analysis revealed the presence of a supposedly operational route for Moco synthesis, as well as of molybdate transporters and molybdoenzymes in the species of the genus *Aquibacillus* and the strains 3ASR75-54^T^, 3ASR75-11^T^, and 3ASR75-286, whereas they were missing for the genera *Radiobacillus* and *Sediminibacillus*. Besides, strains 3ASR75-54^T^, 3ASR75-11^T^, and 3ASR75-286, and possible other members of their genera, are among the few prokaryotes that harbor the molybdate transporter ModABC in the hypersaline soils from Odiel Saltmarshes Natural Area, according to the low genomic information annotated for this transporter in the SMO1 metagenomic dataset.

### 3.7. Mechanisms detected for survival in hypersaline environments

*Salt-in* and *salt-out* strategies are the two main mechanisms in prokaryotes for osmoregulation under salt stress conditions. Haloarchaea (Youssef et al., 2014) and other extremely halophilic bacteria, such as species of the well-known genus *Salinibacter* (Antón et al., 2002), use the *salt-in* mechanism. These organisms commonly present an acidic proteome to avoid the denaturalization of their proteins under high salt concentrations inside the cell (Oren, 2008), but this adaptation can also be found in microorganisms with *salt-out* strategy (Elevi Bardavid and Oren, 2012; Oren, 2013), which is the most extended mechanism in prokaryotes as it allows survival under a wider range of osmotic conditions (Oren, 2008).

**Figure 10:**
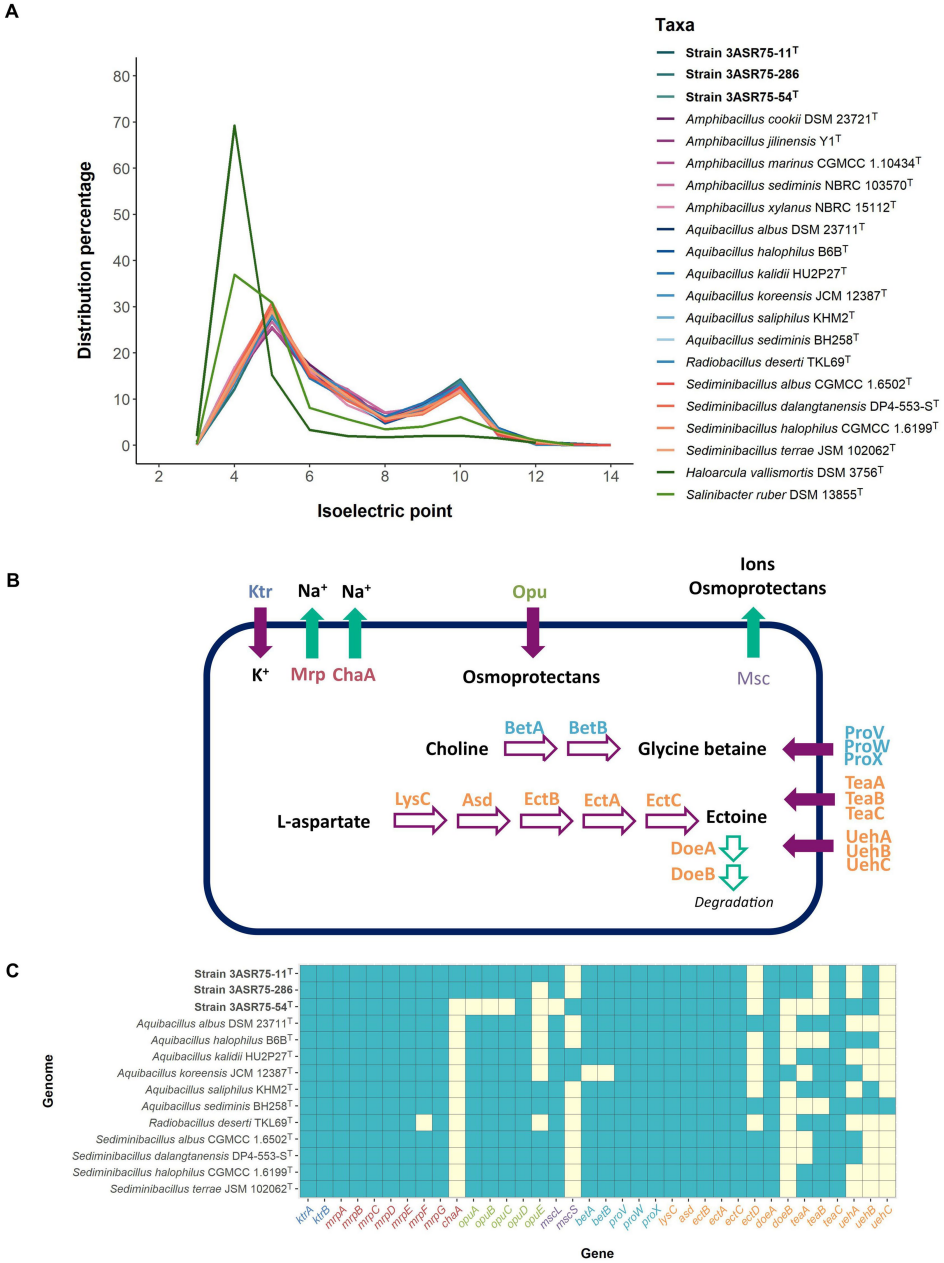
**(A)** Distribution of the isoelectric point of the proteomes under study. *Haloarcula vallismortis* DSM 3756^T^ (GCF_900106715.1) and *Salinibacter ruber* DSM 13855^T^ (GCF_000013045.1) were also included as representatives of extreme halophiles with *salt-in* osmoregulation strategy. The proteomes of members of the family *Bacillaceae* were less acidic than those of the extreme halophiles, suggesting a *salt-out* mechanism. **(B)** Reconstruction of the different strategies employed for the studied strains to cope with osmotic stress. Purple filled arrows indicate uptake, green filled arrows indicate extrusion, purple empty arrows indicate biosynthesis, and green empty arrows indicate degradation. Enzymes and transporters are colored key as follows: K+ uptake (dark blue), Na+ extrusion (red), unspecific osmoprotectant uptake (green), unspecific extrusion (light purple), glycine betaine biosynthesis and uptake (light blue), ectoine metabolism and uptake (orange). **(C)** Heatmap of presence (light blue)/absence (light yellow) of osmoregulation-related genes in the new strains 3ASR75-54^T^, 3ASR75-11^T^, and 3ASR75-286, and the closely related species of the genera *Aquibacillus*, *Radiobacillus*, and *Sediminibacillus*.

The isoelectric profile of strains 3ASR75-54^T^, 3ASR75-11^T^, and 3ASR75-286 (Figure 10A) is similar to that of the species of the genera *Aquibacillus*, *Radiobacillus*, *Sediminibacillus*, and *Amphibacillus*, and differs from the proteome of the extremely halophilic archaeon *Haloarcula vallismortis* and the bacterium *Salinibacter ruber*. This result can be seen as the first evidence that the new isolates could have adopted the *salt-out* mechanism. An insight into the functional annotation of the analyzed genomes of the family *Bacillaceae* pointed out that a sudden increase in the osmotic pressure can be balanced with a cellular uptake of K^+^ through Ktr potassium importers (K03498 and K03499), as it has been previously observed in *Bacillus subtilis* and *Synechocystis* sp. (Holtmann et al., 2003; Zulkifli et al., 2010; Hoffmann and Bremer, 2016). Efflux of Na^+^ is crucial for cell survival due to the toxicity produced by high cytoplasmatic concentration of this ion (Patiño-Ruiz et al., 2022). Mrp multisubunit Na^+^/H^+^ exchangers (K05565, K05566, K05567, K05568, K05569, K05570, and K05571), encoded by the *mrpABCDEFG* operon and detected in our genome dataset, have been shown to provide salt tolerance in *Bacillus subtilis* (Ito et al., 1999), the slight halophile *Halomonas zhaodongensis* (Meng et al., 2014), and the cyanobacteria *Anabaena* sp. and *Synechococcus elongatus* (Blanco-Rivero et al., 2005; Cui et al., 2020), among others. Besides, Mrp antiporters (also known as Sha/Mnh/Pha) seem to contribute to the sporulation process (Kosono et al., 2000; Sun and Shi, 2001; Yoshinaka et al., 2003), which has been observed in the morphology of seven-day-old cells of strains 3ASR75-54^T^, 3ASR75-11^T^, 3ASR75-286, and the species of the genus *Aquibacillus* and *Radiobacillus* (Lee et al., 2006; Zhang et al., 2012, 2015; Amoozegar et al., 2014; Lee and Whang, 2019; Li et al., 2020; Wang et al., 2021; Cho and Whang, 2022). Additionally, the ChaA antiporter (K07300), which has a relevant role in the extrusion of Na^+^ in *Escherichia coli* (Ohyama et al., 1994), has been found in the annotated genome of strains 3ASR75-11^T^ and 3ASR75-286, but not in any of the other studied strains.

Although ion exchange is useful as a first barrier of defense against osmotic pressure, it is more convenient to store compatible solutes into the cytoplasm for prolonged periods of stress (Hoffmann and Bremer, 2016, 2017). There is a diverse spectrum of compatible solutes, but a common characteristic of all of them is their low molecular weight, which allows their accumulation resulting in an increased cytoplasmatic water content without altering the biochemical processes of the prokaryotic cells (Gregory and Boyd, 2021). All the analyzed genomes, except that of strain 3ASR75-54^T^, harbored the osmoprotectant uptake (Opu) system (*opuABC* genes; K05845, K05846, K05847), an ABC transporter for acquisition of different compatible solutes (especially choline) from the environment (Hoffmann and Bremer, 2017; Teichmann et al., 2018) (Figures 10B,C). Besides, all the studied strains presented OpuD from the BCCT (betaine-choline-carnitine-transporter) family, with high affinity for glycine betaine (Kappes et al., 1996), but only 3ASR75-11^T^ together with the species of *Sediminibacillus* and some species of *Aquibacillus* exhibited OpuE from the SSS (sodium-solute-symporter) family of transporters (von Blohn et al., 1997). Another ABC-type transporter, namely ProVWX (K02000, K02001, and K02002), for glycine betaine uptake (Gregory and Boyd, 2021) was present in all genomes under study (Figure 10B).

TeaABC transporter (encoded by *teaABC* genes) (Figure 10C) is a member of the TRAP transporters family with high affinity for ectoine and, to a lesser extent, for its derivative 5-hydroxyectoine (Grammann et al., 2002; Kuhlmann et al., 2008b). It allows the reuptake of secreted ectoine in *Halomonas elongata* and, additionally, plays a role as an effective salvage system for ectoine leaking through the membrane (Kuhlmann et al., 2008b). Previous studies have demonstrated that the presence of the three genes (*teaABC*) is mandatory for the correct function of this ectoine-specific transporter (Grammann et al., 2002). Whereas *teaC* was present in all the studied genomes, *teaA* and *teaB* were only detected in some of them, so, theoretically only the species *A. albus*, *A. kalidii*, *A. saliphilus*, *R. deserti*, *S. halophilus*, and *S. terrae* might have this transporter available. None of the three new isolates encoded the complete set of *teaABC* genes (Figure 10C). UehABC is a second TRAP transporter, previously studied in *Silicibacter pomeroyi* DSS-3, that imports ectoine and hydroxyectoine (Figure 10B). One main difference with TeaABC is that the *uehABC* genes are coregulated with other genes for ectoine degradation. It must be noted that *S. pomeroyi* uses ectoine as sole carbon and nitrogen source, whereas *H. elongata* mainly utilizes it for osmoprotection purposes (Lecher et al., 2009). Only *A. sediminis* harbored the three *uehABC* genes, while they were quite scarce in the other studied genomes (Figure 10C). The higher abundance of TeaABC-related genes over UehABC-related ones might indicate a prevalence of the osmoprotective activity of ectoine over its carbon and nitrogen source utilization in the analyzed strains of the family *Bacillaceae*.

Transportation of ions and compatible solutes into the cytoplasm to balance osmotic pressure must be paired with the ability of the cell to secrete them when a drop in the environmental salt concentration occurs. Msc mechanosensitive channels, which were identified in our genome dataset (K16053, for strain 3ASR75-54^T^; K03282, for the remaining strains), play an important role in releasing ions and organic molecules under osmotic down shock stress (Booth and Blount, 2012; Booth, 2014).

**Figure 11:**
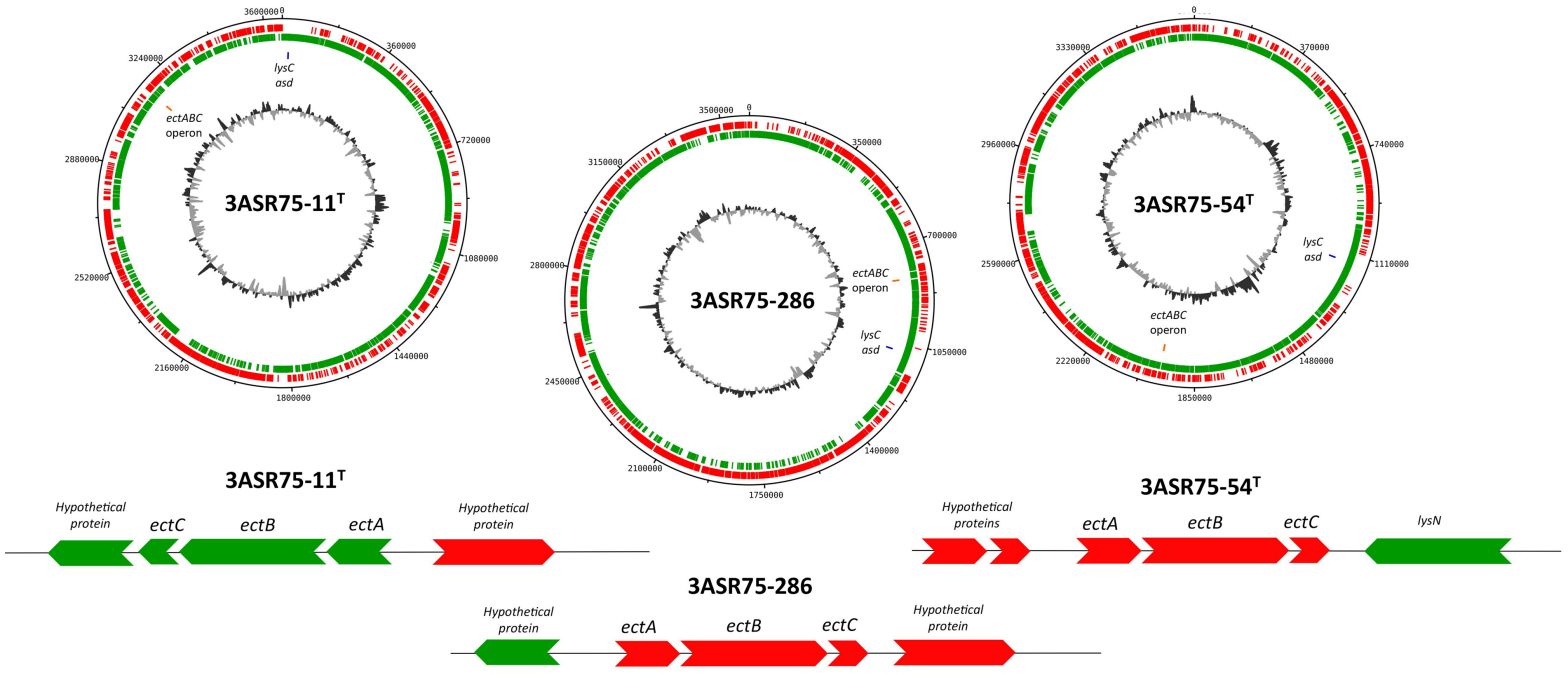
Circular genomic DNA map of strains 3ASR75-11^T^, 3ASR75-286, and 3ASR75-54^T^. Circles indicate, from the inside outward: G + C content; location of the *ectABC* operon (orange) and *lysC* and *asd* genes (blue); coding sequences in the lagging strand (green); coding sequence in the leading strand (red). A closer insight of the *ectABC* operon context is displayed below each genome map.

In addition to the compatible solute uptake, *de novo* biosynthetic potential for the osmolytes glycine betaine and ectoine has been detected in the analyzed genome sequences (Figure 10C). Choline is transformed into glycine betaine in two oxidative steps carried out by BetA (K00108) and BetB (K00130) (Boch et al., 1994), under aerobic conditions (Czech and Bremer, 2018) (Figure 10B). This reaction is widely present in halophilic bacteria and archaea (Gregory and Boyd, 2021) as well as in eukaryotic cells. On the contrary, biosynthesis of ectoine and its derivative is specific to prokaryotes (Czech and Bremer, 2018). Ectoine is obtained from L-aspartate in five steps mediated by aspartate kinase (*lysC*; K00928), aspartate semialdehyde dehydrogenase (*asd*; K00133), diaminobutyrate-2-oxoglutarate transaminase (*ectB*; K00836), L-2,4-diaminobutyric acid acetyltransferase (*ectA*; K06718), and L-ectoine synthase (*ectC*; K06720) (Ono et al., 1999; Figure 10B). All the analyzed genomes possessed the machinery to *de novo* synthesize ectoine. The *ectABC* genes are usually arranged into a single operon (Louis and Galinski, 1997; Kuhlmann et al., 2008a; van Thuoc et al., 2020), as is the case with the studied strains (Figure 11), whereas in other bacterial genomes the synteny and chromosomal placement of the genes is not conserved (León et al., 2018). Some bacteria possess the *ectD* gene that enable the conversion of ectoine into 5-hydroxyectoine, but among the analyzed strains only *A. albus* and *A. halophilus* harbored that gene (Figure 10C). Both osmolytes, ectoine and 5-hydroxyectoine, can be catabolized to be used as carbon and energy sources, albeit this mechanism has not been completely understood. Regardless, DoeA and DoeB proteins seem to be relevant in the degradation (Schwibbert et al., 2011; Reshetnikov et al., 2020). Ectoine producers do not usually catabolize it (Schulz et al., 2017), although some microorganisms can synthesize and consume ectoine, such as *Halomonas elongata* (Schwibbert et al., 2011) and *Sinobaca* sp. (Chen et al., 2022). All the studied genome sequences encoded the ectoine hydrolase gene (*doeA*) and, additionally, *doeB* gene was present in the genome of strains 3ASR75-11^T^ and 3ASR75-286. Thus, organisms that are, at the same time, ectoine producers and consumers may not be as uncommon as previously thought.

After having carried out a comparative genomic analysis we could assert that ectoine plays a significant role in the physiology of strains 3ASR75-54^T^, 3ASR75-11^T^, and 3ASR75-286, as well as in that of their closely related species. Ectoine biosynthetic pathway was completely conserved in the 14 studied genomes. Moreover, two ectoine-targeted transporters were found, one of them (TeaABC) present entirely or with only one gene missing in 11 strains, and the other (UehABC) mostly incomplete for the vast majority of genomes. Besides, only one gene involved in ectoine degradation (*doeA*) was found across all taxa (Figure 10C). Thus, it seems likely that ectoine is mainly utilized as an osmolyte rather than as a source of carbon and nitrogen in our new isolates.

Ectoine is a valuable molecule for biotechnological purposes due to its ability to protect cell components under stressful conditions, such as freezing, high temperature, and drying (Ma et al., 2020; Vandrich et al., 2020). The moderately halophilic bacterium “*Halomonas bluephagenesis*” TD01 has been demonstrated to yield 28 g L^−1^ of ectoine during a 28-h fed-batch growth process (Ma et al., 2020). Besides, ectoine biosynthetic pathway shares the first two steps with the synthesis of one of the most industrially produced amino acids, threonine (Dong et al., 2012). Actually, the aforementioned strain “*H. bluephagenesis*” TD01 is able to produce 33 g L^−1^ of threonine in a 7-liter bioreactor (Du et al., 2020). Strains 3ASR75-54^T^, 3ASR75-11^T^, and 3ASR75-286, as well as the species of the genera *Aquibacillus*, *Radiobacillus*, and *Sediminibacillus*, also harbored the remaining genes that encode the last three steps of threonine biosynthesis: homoserine dehydrogenase (*hom*; K00003), homoserine kinase (*thrB*; K00872), and threonine synthase (*thrC*; K01733). Due to the slow growth rate of the novel isolates, they might not be the preferred source for the biotechnological production of those two molecules. However, they could be of importance to increase their yield after an in-depth study of their ectoine and threonine pathways.

### 3.8. Strategies to thrive in heavy metal contaminated soils

Considering the high concentration of heavy metals detected in the sampled soils, we decided to explore the exporting mechanisms that might be present in the genomes of the new strains and their closest relatives (Supplementary Table S6). The *ars* operon (K03325, K03741, and K03892) can accomplish the reduction of arsenate to arsenite and its posterior expulsion from the cytoplasm (Chauhan et al., 2019; Islam et al., 2022). The presence of those genes in our dataset was expected due to its wide spreading among prokaryotes (Chen et al., 2020). The *zntA* gene (K01534) encoding Zn^2+^/Cd^2+^/Pb^2+^ pumping out coupled to ATP hydrolysis (Rensing et al., 1997; Noll and Lutsenko, 2000) was also identified, but the efficient CzcCBA efflux system for zinc and cadmium was not found (Legatzki et al., 2003). CopA and CopB proteins (K17686 and K01533) are P-type copper efflux transporters that confers resistance to this metal (Rensing et al., 2000; Mana-Capelli et al., 2003) and they were encoded in most of the studied genomes, including the strains 3ASR75-54^T^, 3ASR75-11^T^, and 3ASR75-286. Furthermore, MerB alkylmercury lyase (K00221) was solely found in strain 3ASR75-286. Bacteria can cope with methylmercury contamination thanks to the sequential activity of MerB and MerA proteins, whose expression is controlled by the regulatory protein MerR (Schaefer et al., 2004). However, neither MerA nor MerR were identified in the genome of this strain, which is consistent with the fact that disturbingly high concentrations of methylmercury have not been perceived in the sampled area before (Sainz et al., 2002, 2004).

Multiple copies of *copA*, *copB*, and *zntA* genes have been annotated for the translated CDS of the SMO1 reference metagenomic dataset from a hypersaline soil of Odiel Saltmarshes Natural Area (Supplementary Table S6). Those three genes are among the five more identified KO in the SMO1 dataset, along with a putative transposase (K07496) and a putative ABC transport system permease protein (K02004). The frequency of these genes in the SMO1 metagenome are 2,106, 1,642, and 1,729 copies from a total of 317,837 KO annotated CDS. Therefore, we can assume the importance of the activities of those divalent cation transporters in the metabolism of the prokaryotic population of Odiel soils, including the novel isolates 3ASR75-54^T^, 3ASR75-11^T^, and 3ASR75-286. On the other hand, the arsenic resistant activities seem to be less spread among the prokaryotes inhabiting the soils under study, and none of the annotated functions in the reference SMO1 dataset corresponded to *merB* gene (Supplementary Table S6) which is present exclusively in isolate 3ASR75-286.

### 3.9. Overlooked inhabitants in sampled soils

**Figure 12:**
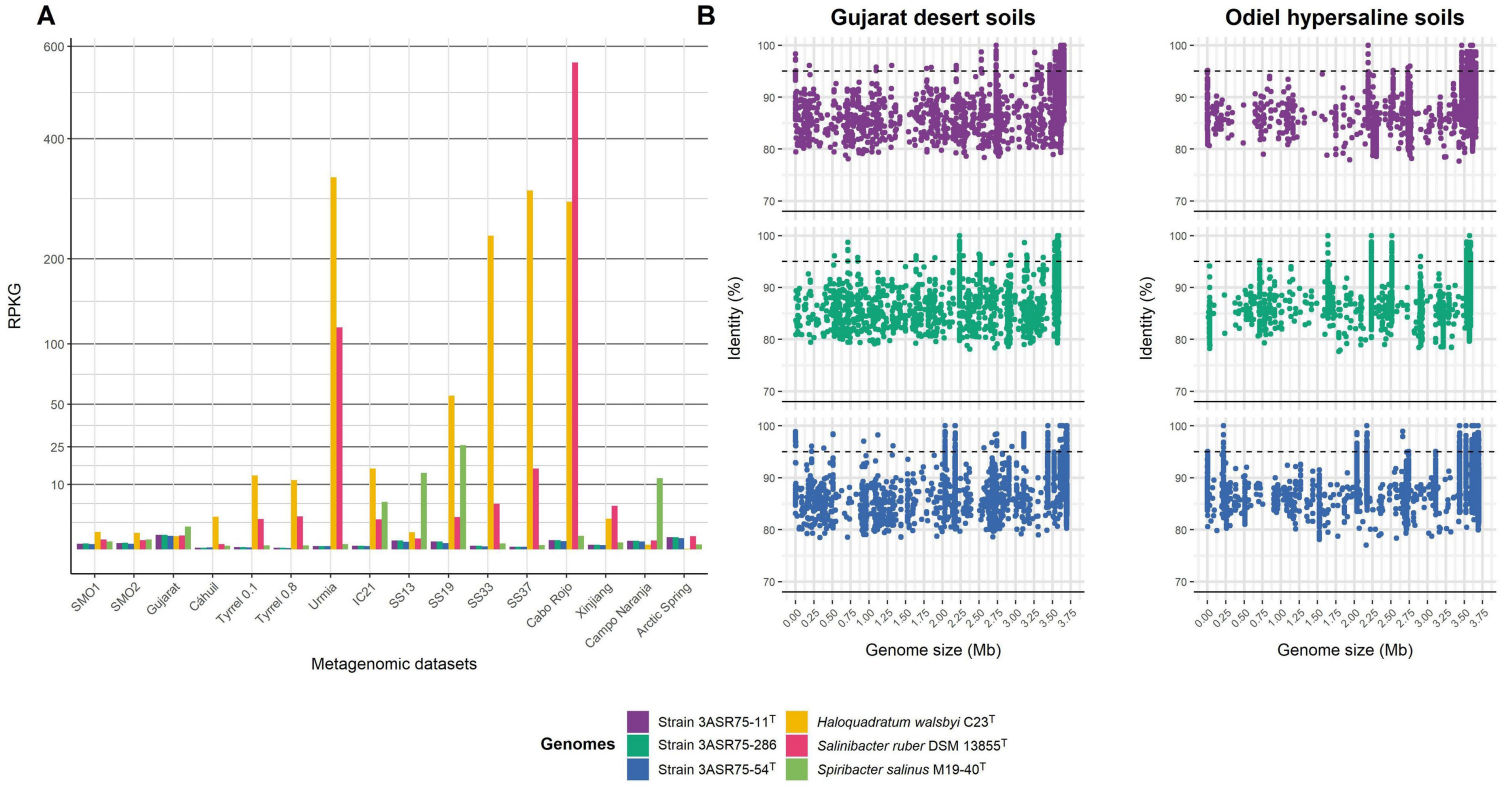
Fragment recruitment of the three new isolated strains from relevant hypersaline metagenomic datasets. Further information about these metagenomes is detailed in Supplementary Table S1. **(A)** Relative abundance represented as RPKG of strains 3ASR75-11^T^, 3ASR75-286, and 3ASR75-54^T^ together to three reference halophilic species. Squared root transformation was performed for Y-axis in order to better visualize low values. **(B)** Plots show read recruitment across genome length of the new isolates from Gujarat desert soil (left) and Odiel Saltmarshes hypersaline soil (right) metagenomes.

Since the ecological distribution of species of *Aquibacillus* and related genera has not been investigated in depth, a fragment recruitment analysis of the new isolated strains was performed from a total of 16 metagenomic libraries originated from hypersaline environments (i.e., saltern ponds with different salt concentrations, hypersaline lakes, saline soils, desert soils, salt crust, microbialites and arctic spring sediments). In order to compare the recruitment results, three representative halophilic microorganisms known to be significantly abundant in saline habitats (*Haloquadratum walsbyi*, *Salinibacter ruber*, and *Spiribacter salinus*) were also included into the analysis. Reads recruitment normalized against the size of the genomes and the database, denoted as RPKG, was low for all the three isolates in the studied metagenomic datasets (Figure 12A). Their abundance was especially rare in environments with (almost-)saturated salt concentration, such as the salterns ponds from Chile (Cáhuil) (Plominsky et al., 2014), Isla Cristina (IC21) (Fernández et al., 2014b), and Santa Pola (SS33 and SS37) (Ghai et al., 2011; Fernández et al., 2014a) in Spain, and Puerto Rico (Cabo Rojo) (Couto-Rodríguez and Montalvo-Rodríguez, 2019), the hypersaline lakes from Australia (Tyrrell 0.1 and Tyrrell 0.8) (Podell et al., 2014) and Iran (Urmia) (Kheiri et al., 2023), and the salt crust from the Qi Jiao Jing Lake in China (Xinjiang) (Xie et al., 2022), and slightly higher at intermediate salinities [SS13 and SS19 from Santa Pola salterns (Ghai et al., 2011; Fernández et al., 2014a), and microbialites from Campo Naranja in Argentina (Perez et al., 2020)]. Recruitment plot from SMO1 and SMO2 metagenomes (Vera-Gargallo et al., 2018), corresponding to samples collected a few years ago from the same hypersaline soils (Odiel) than those analyzed in the present study, displayed similar abundance of the new isolates to that found for intermediate salterns ponds (SS13 and SS19). Furthermore, previous taxonomic annotation of SMO1 and SMO2 databases registered that the phylum *Bacillota* (to which the new strains belong) represented a small fraction of the microbial population (Vera-Gargallo et al., 2019). Indeed, relative abundances for strains 3ASR75-11^T^ and 3ASR75-286 varied between 0.02–0.0182% whereas strain 3ASR75-54^T^ was even less abundant, with 0.0163–0.0157%. Soils from Gujarat desert, which are mostly dominated by *Pseudomonadota* (Patel et al., 2015), and hypersaline Arctic Spring sediments, whose microbial life has been hypothesized as survival organisms on Mars (Magnuson et al., 2022), harbored the highest abundance for our isolates (Figure 12A), but with values ranging from 0.0416–0.0364% and 0.048–0.042%, respectively. In all cases, these values were lower than the 0.1% threshold commonly used to label the so-called “rare biosphere” (Pedrós-Alió, 2012). Therefore, strains 3ASR75-54^T^, 3ASR75-11^T^, and 3ASR75-286 could be considered part of the low-abundant prokaryotic fraction inhabiting soils from Odiel Saltmarshes Natural Area. A closer look at the recruitments to visualize up to what extent the genome of the new strains is covered in SMO1 and Gujarat metagenomes showed many coverage gaps over the 95% identity, a widely accepted cutoff for species delineation (Figure 12B). Again, this finding suggests that the new species represented by strain 3ASR75-54^T^ and by strains 3ASR75-11^T^ and 3ASR75-286, respectively, are scarce inhabitants of the studied soils, although they have demonstrated to be relatively easy to cultivate and manipulate in laboratory conditions. Our work emphasizes the relevance of the traditional isolation and characterization methodology to explore the rare biosphere. Previous research to uncover the culturable diversity of the sampled environment succeeded to isolate a new member of the phylum *Balneolota* (Galisteo et al., 2023), which has been listed as one of the major phyla in the hypersaline soils of Odiel Saltmarhes Natural Area (Vera-Gargallo et al., 2019). In the authors’ opinion, culture-dependent studies are indispensable for a better knowledge of the microbial diversity unveiled by metagenomic approaches but also for discovering taxa that cannot be detected by high throughput sequencing.

## 4. Conclusion

A large set of strains affiliated to the family *Bacillaceae* was isolated in this study after a long incubation period and further divided into two separate groups. Representative strains of these groups were selected for phylogenomic, comparative genomic, phenotypic, and chemotaxonomic analyses, which confirmed their placement as new bacterial species. Besides, AAI values between one of these taxa and the species of the closely related genera *Aquibacillus*, *Radiobacillus*, and *Sediminibacillus* acknowledged its description as a new genus. Thus, we propose the classification of strain 3ASR75-54^T^ within the genus *Aquibacillus,* as a new species, for which the name *Aquibacillus salsiterrae* sp. nov. is proposed, and the placement of strains 3ASR75-11^T^ and 3ASR75-286 into a new separate genus and species, for which the new name *Terrihalobacillus insolitus* gen. nov., sp. nov. is proposed. The descriptions of these new taxa are shown below.

The new species of the genus *Aquibacillus* and the new genus *Terrihalobacillus* encoded the well-conserved molybdenum cofactor biosynthetic pathway and the molybdenum-dependent enzymes, differentiating them from other closely related member of the family *Bacillaceae*, such as the genera *Radiobacillus* and *Sediminibacillus*. Besides, in-depth in-silico analysis of their genome sequences revealed strategies to deal with high salt concentration and heavy metal contamination of the soils from Odiel Saltmarshes Natural Area. The *salt-out* mechanism of osmoregulation seems to be prevailing in the three new isolates, harboring proteins for uptake and *de novo* biosynthesis of ectoine and glycine betaine, two of the most frequent compatible solutes in prokaryotes. The genes *arsC*, *arsR*, *arsB*, *zntA*, *copA*, and *copB*, among others, have been identified in the genomes of the isolates, pointing at their tolerance to heavy metals, such as arsenic, zinc, cadmium, lead, and copper. The abundance of the new described species from the genera *Aquibacillus* and *Terrihalobacillus* is extremely low in all the studied hypersaline environments, including the isolation area, with a relative abundance under 0.1%, the threshold for the so-called “rare-biosphere.”

### Description of *Aquibacillus salsiterrae* sp. nov.

*Aquibacillus salsiterrae* sp. nov. (sal.si.ter’rae. L. masc. adj. *salsus* salty; L. fem. n. *terra* earth, soil; N.L. gen. n. *salsiterrae* of salty soil).

Cells are Gram-stain-positive, motile rods with a size of 0.7–1.0 × 2.8–3.0 μm. Endospores are formed at terminal position. Colonies are semi-translucent and white-colored, with a size of 2.0–2.5 mm when grown in R2A medium supplemented with 7.5% (w/v) salts after 24 h of incubation at 37°C. Facultative anaerobe. The temperature range for growth is 11–45°C (optimum at 37°C). The pH values supporting growth are 6.0–8.0 (optimum at pH 6.0) and the NaCl concentration for growth is 0.5–17% (w/v) [optimum at 7% (w/v)]. Catalase and oxidase positive. Hydrolyzes aesculin but not casein, DNA, gelatin, starch, and Tween 80. Reduces nitrate and nitrite. Positive for methyl red test but negative for Voges-Proskauer test, meaning that it uses the mixed-acid pathway for glucose fermentation. Indole production, Simmons’ citrate test, phenylalanine deaminase, urease, and H_2_S production are negative. Acids are produced from D-arabinose, D-fructose, glycerol, D-glucose, lactose, maltose, mannitol, sucrose, D-trehalose, and D-xylose, but not from D-galactose. Utilizes L-arabinose, D-cellobiose, D-maltose, D-mannose, melibiose, D-trehalose, D-xylose, butanol, dulcitol, ethanol, glycerol, mannitol, methanol, propranolol, D-sorbitol, xylitol, benzoate, formate, fumarate, hippurate, malate, and propionate as sole source of carbon and energy, but not aesculin, amygdalin, D-melezitose, ribose, starch, acetate, butyrate, glutamate, pyruvate, and valerate. Utilizes L-alanine, L-asparagine, aspartic acid, L-cysteine, glycine, L-glutamine, L-methionine, ornithine, L-phenylalanine, L-serine, L-threonine, tryptophane, and valine as sole source of carbon, nitrogen, and energy, but not arginine. Major fatty acids are anteiso-C_15:0_, followed by iso-C_15:0_ and anteiso-C_17:0_. The genome of the type strain has a G + C content of 38.0 mol% and its approximate size is 3.70 Mb.

The type strain, 3ASR75-54^T^ (=CCM 9168^T^ = CECT 30368^T^), was isolated from a hypersaline soil at the Odiel Saltmarshes Natural Area in Huelva (Southwest Spain). The accession number for the 16S rRNA gene sequence is ON652841 and that for the genome sequence is GCF_028416595.1.

### Description of *Terrihalobacillus* gen. nov.

*Terrihalobacillus* gen. nov. (Ter.ri.ha.lo.ba.cil’lus. L. fem. n. *terra*, land; Gr. masc. n. *hals,* salt; L. masc. dim. n. *bacillus,* a small rod; N.L. masc. n. *Terrihalobacillus,* a small rod from salty land).

Cells are Gram-stain-positive, motile, and endospore-forming rods that form white-pigmented colonies. Endospores are formed at terminal position. Moderately halophilic, growing in a wide range of NaCl concentrations. Mesophile and facultative anaerobe. Catalase and oxidase positive. Genome mining reveals the biosynthetic pathway for the molybdenum cofactor and genes encoding for molybdoenzymes. Major fatty acid is anteiso-C_15:0_. It belongs to the family *Bacillaceae*, order *Caryophanales*, class *Bacilli*, and phylum *Bacillota*. The DNA G + C content is 38.0–38.1 mol% (genome). The type species is *Terrihalobacillus insolitus*.

### Description of *Terrihalobacillus insolitus* sp. nov.

*Terrihalobacillus insolitus* sp. nov. (in.so’li.tus. L. masc. adj. *insolitus*, unusual or uncommon).

Cell are Gram-stain-positive, motile rods with a size of 0.5–1.0 × 3.0–5.0 μm. Endospores are formed at terminal position. Colonies are circular, convex, opaque, and white-colored, with a size of 1 mm when growing in R2A medium supplemented with 7.5% (w/v) salts after 24 h of incubation at 37°C. Facultative anaerobe. The temperature range for growth is 10–45°C (optimum at 37°C). The pH values supporting growth are 4.0–9.0 (optimum at pH 5.0) and the NaCl concentration for growth is 0.5–20% (w/v) [optimum at 2% (w/v)]. Catalase and oxidase positive. Does not hydrolyze aesculin, casein, DNA, gelatin, starch, and Tween 80. Reduces nitrate, but not nitrite. Positive for the methyl red test but negative for the Voges-Proskauer test, meaning that it uses the mixed-acid pathway for glucose fermentation. Indole production, Simmons’ citrate test, phenylalanine deaminase, urease, and H_2_S production are negative. Acids are produced from D-fructose, D-galactose, D-glucose, glycerol, lactose, maltose, mannitol, sucrose, and D-trehalose, but not from D-arabinose. Utilizes D-fructose, D-maltose, D-mannose, D-melezitose, salicin, sucrose, D-trehalose, D-xylose, mannitol, xylitol, benzoate, butyrate, fumarate, hippurate, malate, and pyruvate as sole source of carbon and energy, but not aesculin, amygdalin, D-cellobiose, D-galactose, D-glucose, D-lactose, D-raffinose, starch, butanol, acetate, glutamate, and valerate. Utilizes glycine as sole source of carbon, nitrogen, and energy source, but not L-asparagine, aspartic acid, and L-threonine. Major fatty acid is anteiso-C_15:0_. The genome of the type strain has a G + C content of 38.0 mol% and its approximate size is 3.66 Mb.

The type strain, 3ASR75-11^T^ (=CCM 9167^T^ = CECT 30367^T^), was isolated from a hypersaline soil at the Odiel Saltmarshes Natural Area in Huelva (Southwest Spain). The accession number for the 16S rRNA gene sequence is ON652838 and that for the genome sequence is GCF_028416575.1. Strain 3ASR75-286 is an additional strain of this species. Its DNA G + C content is 38.1 mol% (genome) and its approximate genome size is 3.59 Mb. The accession number for its 16S rRNA gene sequence is ON653021 and that for its genome sequence is GCF_028416555.1.

## Data availability statement

The datasets presented in this study can be found in online repositories. The names of the repository/repositories and accession number(s) can be found in the article/Supplementary material.

## Author contributions

AV and CS-P conceived the study. CG, CS-P, and AV obtained the environmental samples. CG accomplished the laboratory experiments and the in-silico analysis, supported by CS-P and RRH, respectively. CG drafted the manuscript. CG, CS-P, RRH, and AV revised the manuscript. All authors contributed to the article and approved the submitted version.

## Funding

This study was supported by grant PID2020-118136GB-I00 funded by MCIN/AEI/10.13039/501100011033 (to AV and CS-P). AV acknowledges the support from the Junta de Andalucía (grants P20_01066 and BIO-213), all with FEDER funds. CG was a recipient of a predoctoral fellowship (PRE2018-083242) from the Spanish Ministry of Science and Innovation. RRH was a recipient of a short-stay grant (PRX21/00598) from the Spanish Ministry of Universities.

## Conflict of interest

The authors declare that the research was conducted in the absence of any commercial or financial relationships that could be construed as a potential conflict of interest.

## Publisher’s note

All claims expressed in this article are solely those of the authors and do not necessarily represent those of their affiliated organizations, or those of the publisher, the editors and the reviewers. Any product that may be evaluated in this article, or claim that may be made by its manufacturer, is not guaranteed or endorsed by the publisher.

## Footnotes

^1^https://www.ezbiocloud.net

^2^https://blast.ncbi.nlm.nih.gov

^3^https://github.com/cristinagalisteo/gitana
