# Supplementary material for: A step into the rare biosphere: genomic features of the new genus Terrihalobacillus and the new species Aquibacillus salsiterrae from hypersaline soils
Source: Front Microbiol. 2023 May 9;14:1192059. doi: 10.3389/fmicb.2023.1192059 (PMC10203224; doi:10.3389/fmicb.2023.1192059)
Supplement: Supplementary file 1 [file Data_Sheet_1.pdf]

# *Supplementary Material*

# A step into the rare biosphere: genomic features of the new genus *Terrihalobacillus* and the new species *Aquibacillus salsiterrae* from hypersaline soils

**Cristina Galisteo, Rafael R. de la Haba, Cristina Sánchez-Porro* and Antonio Ventosa***

*** Correspondence:** ventosa@us.es; sanpor@us.es

## 1 Supplementary Tables

**Table S1.** Metadata associated with the metagenomic datasets used for the fragment recruitment analyses. NA, not available.

| Metagenomic dataset | Sample | Salt concentration | SRA accession number | Reference |
|---|---|---|---|---|
| SMO1 | Hypersaline soil (Huelva, Spain) | 24.0 dS/m | SRR5753725 | Vera Gargallo et al. (2018) |
| SMO2 | Hypersaline soil (Huelva, Spain) | 54.4 dS/m | SRR5753724 | Vera Gargallo et al. (2018) |
| Gujarat | Saline desert (Kutch, India) | NA | ERP005612 | Patel et al. (2015) |
| Cáhuil | Saltern crystallizer (Cáhuil, Chile) | 34 % (w/v) salts | SRR1549536 | Plominsky et al. (2014) |
| Tyrrell 0.1 | Hypersaline lake (Victoria, Australia) | 29% (w/v) salts | SRR5637210 | Podell et al. (2014) |
| Tyrrell 0.8 | Hypersaline lake (Victoria, Australia) | 29% (w/v) salts | SRR5637211 | Podell et al. (2014) |
| Urmia | Hypersaline lake (Iran) | 510.3 dS/m | SRR19434976 | Kheiri et al. (2023) |
| IC21 | Saltern pond (Isla Cristina, Spain) | 21 % (w/v) salts | SRR988245 | Fernández et al. (2014b) |
| SS13 | Saltern pond (Alicante, Spain) | 13 % (w/v) salts | SRR944625 | Fernández et al. (2014a) |
| SS19 | Saltern pond (Alicante, Spain) | 19 % (w/v) salts | SRR328982 | Ghai et al. (2011) |
| SS33 | Saltern pond (Alicante, Spain) | 33 % (w/v) salts | SRR979792 | Fernández et al. (2014a) |
| SS37 | Saltern pond (Alicante, Spain) | 37 % (w/v) salts | SRR328983 | Ghai et al. (2011) |

| | | | | |
|---|---|---|---|---|
| Cabo Rojo | Solar saltern (Puerto Rico) | 34 % (w/v) salts | SRR8816319 | Couto-Rodríguez and Montalvo-Rodríguez (2019) |
| Xinjiang | Salt crust (Xinjiang, China) | > 30 % (w/v) salts | SRR18572989 | Xie et al. (2022) |
| Campo Naranja | Microbialites (Catamarca, Argentina) | 17.8 dS/m | ERR3083899 | Perez et al. (2020) |
| Arctic Spring | Hypersaline artic spring sediments | 271.6 (g/L) Total dissolved solids | SRR13628066 | Magnuson et al. (2022) |

**Table S2.** Main genomic features of the representative strains isolated in this study and the type strains of species of the genera *Amphibacillus*, *Aquibacillus*, *Radiobacillus*, and *Sediminibacillus* used for the comparative genomic analyses.

| Strain | Assembly accession number | Size (bp) | Contigs | N50 | G+C (mol%) | Completeness (%) | Contamination (%) | CDS | rRNA | tRNA | CRISPRs |
|---|---|---|---|---|---|---|---|---|---|---|---|
| **3ASR75-54$^T$** | GCF_028416595.1 | 3,700,068 | 70 | 225,253 | 37.98 | 99.34 | 0.99 | 3535 | 15 | 104 | 1 |
| **3ASR75-11$^T$** | GCF_028416575.1 | 3,660,390 | 71 | 172,976 | 38.03 | 98.68 | 1.16 | 3590 | 9 | 66 | 4 |
| **3ASR75-286** | GCF_028416555.1 | 3,592,467 | 67 | 115,843 | 38.14 | 98.68 | 0.33 | 3617 | 9 | 66 | 0 |
| *Amphibacillus cookie* DSM 23721$^T$ | GCF_016908375.1 | 3,820,199 | 29 | 312,532 | 37.27 | 99.34 | 0.66 | 3492 | 8 | 50 | 3 |
| *Amphibacillus jilinensis* Y1$^T$ | GCF_000306965.1 | 3,836,603 | 30 | 676,113 | 37.27 | 99.34 | 0.88 | 3570 | 3 | 55 | 0 |
| *Amphibacillus marinus* CGMCC 1.10434$^T$ | GCF_900110345.1 | 3,468,523 | 29 | 227,437 | 38.57 | 99.34 | 1.66 | 3274 | 5 | 46 | 0 |
| *Amphibacillus sediminis* NBRC 103570$^T$ | GCF_001552275.1 | 3,447,472 | 74 | 116,850 | 38.12 | 99.34 | 1.77 | 3083 | 7 | 57 | 4 |
| *Amphibacillus xylanus* NBRC 15112$^T$ | GCF_000307165.1 | 2,569,486 | 1 | 2,569,486 | 35.72 | 98.68 | 0.22 | 2402 | 18 | 56 | 0 |
| *Aquibacillus albus* DSM 23711$^T$ | GCF_016908325.1 | 4,408,260 | 61 | 143,678 | 36.67 | 98.68 | 2.65 | 4128 | 13 | 109 | 0 |
| *Aquibacillus halophilus* B6B$^T$ | GCF_009649745.1 | 4,324,655 | 56 | 226,090 | 35.9 | 99.34 | 1.66 | 4151 | 13 | 91 | 0 |
| *Aquibacillus kalidii* HU2P27$^T$ | GCF_014280935.1 | 4,255,198 | 66 | 110,348 | 36.01 | 99.34 | 1.99 | 4126 | 8 | 69 | 0 |
| *Aquibacillus koreensis* JCM 12387$^T$ | GCF_028416535.1 | 4,328,474 | 56 | 190,688 | 36.82 | 98.68 | 0.55 | 4227 | 7 | 69 | 0 |
| *Aquibacillus saliphilus* KHM2$^T$ | GCF_020404745.1 | 4,225,206 | 47 | 183,641 | 35.7 | 99.34 | 1.99 | 4168 | 13 | 91 | 0 |

| | | | | | | | | | | | |
|---|---|---|---|---|---|---|---|---|---|---|---|
| *Aquibacillus sediminis* BH258$^T$ | GCF_005870085.1 | 4,381,979 | 85 | 135,569 | 37.35 | 99.34 | 1.77 | 4146 | 18 | 153 | 1 |
| *Radiobacillus deserti* TKL69$^T$ | GCF_007301515.1 | 3,615,398 | 1 | 3,615,398 | 38.53 | 99.34 | 0.44 | 3786 | 24 | 67 | 0 |
| *Sediminibacillus albus* CGMCC 1.6502$^T$ | GCF_900101125.1 | 3,565,081 | 14 | 634,970 | 41.27 | 99.34 | 1.21 | 3505 | 9 | 60 | 1 |
| *Sediminibacillus dalangtanensis* DP4-553-S$^T$ | GCF_017792025.1 | 4,100,703 | 1 | 4,100,703 | 43.55 | 99.34 | 5.97 | 3948 | 27 | 72 | 0 |
| *Sediminibacillus halophilus* CGMCC 1.6199$^T$ | GCF_900103695.1 | 4,149,758 | 13 | 756,573 | 42.86 | 99.34 | 1.21 | 4054 | 8 | 59 | 0 |
| *Sediminibacillus terrae* JSM 102062$^T$ | GCF_009602435.1 | 4,093,757 | 29 | 486,406 | 43.99 | 99.34 | 1.21 | 3985 | 11 | 67 | 0 |

**Table S3.** Fatty acid composition (%) of strains 3ASR75-54$^{T}$ and 3ASR75-11$^{T}$ and related species of the genera *Aquibacillus*, *Radiobacillus*, and *Sediminibacillus*.
1. Strain 3ASR75-54$^{T}$; 2. Strain 3ASR75-11$^{T}$; 3. *Aquibacillus albus* YIM 93624$^{T}$; 4. *Aquibacillus halophilus* B6B$^{T}$; 5. *Aquibacillus kalidii* HU2P27$^{T}$; 6. *Aquibacillus koreensis* BH30097$^{T}$; 7. *Aquibacillus salifodinae* WSY08-1$^{T}$; 8. *Aquibacillus saliphilus* KHM2$^{T}$; 9. *Aquibacillus sediminis* BH258$^{T}$; 10. *Radiobacillus deserti* TKL69$^{T}$; 11. *Sediminibacillus halophilus* EN8d$^{T}$. Major fatty acids (above 10.0%) are highlighted in bold. Fatty acids representing below 1.0% for all the strains were omitted. -, not detected; tr, < 1.0%.

| **Fatty acid** | **1** | **2** | **3**[a] | **4**[b] | **5**[c] | **6**[d] | **7**[e] | **8**[f] | **9**[g] | **10**[h] | **11**[i] |
|---|---|---|---|---|---|---|---|---|---|---|---|
| $C_{14:0}$ | 2.2 | 1.2 | 1.1 | 1.0 | 2.3 | 1.8 | tr | - | - | 1.4 | 0.6 |
| $C_{15:0}$ | - | - | | - | - | 5.1 | - | - | - | - | - |
| $C_{16:0}$ | 5.5 | 5.1 | **13.9** | 2.8 | 9.3 | 5.9 | 1.1 | 1.47 | 4.9 | **10.4** | 4.3 |
| $C_{16:0}$ N alcohol | - | - | 2.7 | - | - | - | - | - | - | - | - |
| $C_{18:0}$ | 1.1 | - | - | - | - | - | - | - | - | 2.1 | 0.7 |
| iso-$C_{14:0}$ | 3.9 | 5.3 | 1.5 | 3.4 | 2.3 | 8.3 | 3.1 | 4.7 | 6.4 | 1.7 | tr |
| iso-$C_{14:0}$ E | - | - | - | - | 7.4 | - | - | - | - | - | - |
| iso-$C_{15:0}$ | **11.6** | 5.4 | 4.1 | 5.3 | **14.9** | 6.1 | 4.1 | 4.7 | **10.1** | **24.0** | 5.4 |
| iso-$C_{16:0}$ | 6.2 | 6.8 | 9.9 | 4.2 | 4.8 | **14.4** | 3.3 | 4.0 | **12.7** | 5.3 | 3.9 |
| iso-$C_{17:0}$ | 1.5 | - | 1.1 | - | 2.4 | - | - | tr | - | 2.2 | tr |
| iso $C_{17:1}$ $\omega 5c$ | - | - | - | - | - | - | - | - | - | 2.4 | - |
| anteiso-$C_{15:0}$ | **47.9** | **66.4** | **52.1** | **75.1** | **27.9** | **43.4** | **72.7** | **57.5** | **49.2** | **30.0** | **59.0** |
| anteiso-$C_{17:0}$ | **10.7** | 9.9 | 5.2 | 4.4 | 7.8 | 7.0 | 1.0 | **16.4** | **10.9** | 8.7 | **25.0** |
| $C_{15:1}$ $\omega 5c$ | - | - | - | - | 6.1 | - | - | - | - | 2.3 | - |
| $C_{15:1}$ $\omega 7c$ alcohol | - | - | - | - | - | - | - | - | 2.5 | - | - |
| $C_{16:1}$ $\omega 5c$ | - | - | - | - | 2.8 | - | - | - | - | - | - |
| $C_{16:1}$ $\omega 7c$ alcohol | 4.6 | - | - | - | 1.6 | 3.6 | 1.9 | 4.9 | - | 1.6 | - |
| $C_{16:1}$ $\omega 11c$ | 4.9 | - | 2.3 | - | 6.4 | 2.2 | - | tr | 1.0 | 2.6 | - |
| $C_{20:4}$ $\omega 6,9,12,15c$ | - | - | - | - | - | - | - | - | - | 1.9 | - |
| $C_{16:1}$ $\omega 6c$ and/or $C_{16:1}$ $\omega 7c$ | - | - | 1.6 | - | - | - | - | - | - | - | - |
| $C_{18:1}$ $\omega 6c$ and/or $C_{18:1}$ $\omega 7c$ | - | - | 1.5 | - | - | - | - | - | - | - | - |

[a] Zhang et al. (2012); [b] Amoozegar et al. (2014); [c] Wang et al (2021); [d] Lee et al. (2006); [e] Zhang et al. (2015); [f] Cho and Whang (2022); [g] Lee and Whang (2019); [h] Li et al. (2020); [i] Carrasco et al. (2008).

**Table S4.** Differential biochemical features of the novel strains and representative members of the genera *Aquibacillus* and *Radiobacillus*.

1. Strain 3ASR75-54$^T$; 2. Strain 3ASR75-11$^T$; 3. Strain 3ASR75-286; 4. *Aquibacillus albus* JCM 17364$^T$; 5. *Aquibacillus koreensis* JCM 12387$^T$; 6. *Aquibacillus salifodinae* JCM 19761$^T$; 7. *Radiobacillus deserti* TKL69$^T$.

All strains were unable to hydrolyze casein, DNA, gelatin, starch, and Tween 80. They all tested negative for indole production, Voges-Proskauer, Simmons' citrate, phenylalanine deaminase, and $H_2S$ production, and positive for methyl red test and nitrate reduction. All strains were able to produce acid from glycerol, D-glucose, and maltose. They all utilized D-xylose as sole source of carbon and energy, but not starch, acetate, glutamate, and valerate. Data for hydrolysis of casein and DNA, indole and $H_2S$ production, methyl red and Vogues-Proskauer tests, Simmons' citrate, phenylalanine deaminase, nitrate reduction, acid production from glycerol, D-glucose, and maltose, and utilization of starch, D-xylose, acetate, citrate, glutamate, and valerate as sole carbon and energy sources of *Radiobacillus deserti* TKL69$^T$ are not available.

+, positive; -, negative; NA, data not available; w, weakly positive.

| **Characteristic** | **1** | **2** | **3** | **4** | **5** | **6** | **7[a]** |
|---|---|---|---|---|---|---|---|
| Aesculin hydrolysis | + | - | - | + | + | + | NA |
| Urease | - | - | - | + | + | + | - |
| Nitrite reduction | + | - | - | + | - | - | NA |
| **Acid production from carbohydrates:** | | | | | | | |
| D-arabinose | + | - | - | + | - | - | NA |
| D-fructose | + | + | + | + | - | + | NA |
| D-galactose | - | + | + | + | + | - | NA |
| Lactose | + | + | + | + | + | - | NA |
| Mannitol | + | + | + | - | + | - | NA |
| Sucrose | + | + | + | + | + | - | NA |
| D-trehalose | + | + | + | + | - | - | NA |
| D-xylose | + | - | + | + | + | + | NA |
| **Utilization as sole carbon and energy source of:** | | | | | | | NA |
| Aesculin | - | - | - | - | + | w | NA |
| Amygdalin | - | - | - | w | + | - | NA |
| L-arabinose | + | + | - | + | + | + | NA |
| D-cellobiose | + | - | - | + | + | w | + |
| D-fructose | w | + | + | + | + | w | NA |
| D-galactose | w | - | - | + | w | - | + |
| D-glucose | w | - | - | w | w | - | NA |
| D-lactose | w | - | - | w | + | - | NA |
| D-maltose | + | + | + | - | + | - | + |
| D-mannose | + | + | + | + | - | + | + |
| Melibiose | + | w | - | + | - | + | NA |
| D-melezitose | - | + | w | + | - | + | NA |
| Ribose | - | + | - | + | - | + | NA |
| D-raffinose | w | - | - | w | - | + | NA |
| Salicin | w | + | w | + | + | - | NA |
| Sucrose | NA | + | + | - | + | - | NA- |

| | | | | | | | |
|---|---|---|---|---|---|---|---|
| D-trehalose | + | + | + | + | + | w | NA |
| Butanol | + | - | - | + | - | + | NA |
| Dulcitol | + | w | - | + | + | + | NA |
| Ethanol | + | + | - | + | + | + | NA |
| Glycerol | + | w | - | + | + | + | NA |
| Mannitol | + | + | + | - | - | + | NA |
| Methanol | + | + | - | - | - | - | NA |
| Propranolol | + | + | - | - | - | - | NA |
| D-sorbitol | + | + | - | + | - | + | NA |
| Xylitol | + | w | + | - | - | - | NA |
| Benzoate | + | + | + | + | - | w | NA |
| Butyrate | - | + | w | + | - | + | NA |
| Formate | + | NA | NA | + | w | + | NA |
| Fumarate | + | + | + | + | w | + | NA |
| Hippurate | + | + | + | + | - | + | NA |
| Malate | + | + | + | + | - | + | NA |
| Pyruvate | - | + | + | + | - | + | NA |
| Propionate | + | w | w | - | + | w | NA |
| **Utilization as sole carbon, nitrogen, and energy source of:** | | | | | | | |
| L-alanine | + | + | - | + | - | + | - |
| Arginine | - | + | - | + | - | + | NA |
| L-asparagine | + | - | - | + | - | + | NA |
| Aspartic acid | + | - | - | + | - | + | NA |
| L-Cysteine | + | + | - | + | - | + | - |
| Glycine | + | + | + | + | - | + | NA |
| L-glutamine | + | + | - | w | - | + | NA |
| L-isoleucine | w | + | - | w | - | + | NA |
| Lysine | w | w | - | + | - | + | NA |
| L-methionine | + | + | - | + | - | + | NA |
| Ornithine | + | + | - | + | - | - | NA |
| L-phenylalanine | + | + | - | + | w | + | NA |
| L-serine | + | + | - | + | - | - | NA |
| L-threonine | + | - | - | + | - | - | NA |
| Tryptophane | + | w | - | + | - | + | NA |
| Valine | + | + | - | + | - | + | NA |

[a] Li et al. (2020).

**Table S5.** KEGG functional Orthologs (KO) identifiers associated with the molybdenum cofactor biosynthetic pathway detected in the new strains and relatives of the genera *Aquibacillus*, *Radiobacillus*, and *Sediminibacillus*. The number of copies for each gene identified in the metagenomic dataset SMO1 are indicated in the last column.
1. Strain 3ASR75-11$^T$, 2. Strain 3ASR75-286, 3. Strain 3ASR75-54$^T$, 4. *A. albus* DSM 23711$^T$; 5. *A. halophilus* B6B$^T$; 6. *A. kalidii* HU2P27$^T$; 7. *A. koreensis* JCM 12387$^T$; 8. *A. saliphilus* KHM2$^T$; 9. *A. sediminis* BH258$^T$; 10. *R. deserti* TKL69$^T$; 11. *S. albus* CGMCC 1.6502$^T$; 12. *S. dalangtanensis* DP4-553-S$^T$; 13. *S. halophilus* CGMCC 1.6199$^T$; 14. *S. terrae* JSM 102061$^T$. P, KO identifier present; A, KO identifier absent.

| Gene | KO identifier | Function | 1 | 2 | 3 | 4 | 5 | 6 | 7 | 8 | 9 | 10 | 11 | 12 | 13 | 14 | SMO1 copies |
|---|---|---|---|---|---|---|---|---|---|---|---|---|---|---|---|---|---|
| *moaA* | K03639 | Step 1: Transforms 5'-GTP into cPMP | P | P | P | P | P | P | P | P | P | A | A | A | A | A | 151 |
| *moaB* | K03638 | Unknown function | P | P | P | P | P | P | P | P | P | A | A | A | A | A | 43 |
| *moaC* | K03637 | Step 1: Transforms 5'-GTP into cPMP | P | P | P | P | P | P | P | P | P | A | A | A | A | A | 48 |
| *moaD* | K03636 | Step 2: Inserts two sulfurs atom into cPMP | P | P | P | P | P | P | P | P | P | A | A | A | A | A | 84 |
| *moaE* | K03635 | Step 2: Inserts two sulfurs atom into cPMP | P | P | P | P | P | P | P | P | P | A | A | A | A | A | 62 |
| *mobA* | K03752 | Step 4: Generates bis-MGD | P | P | P | P | P | P | P | P | P | A | A | P | P | P | 38 |
| *mobB* | K03753 | Unknown function | P | P | P | P | P | P | P | P | P | A | A | A | A | A | 3 |
| *mocA* | K07141 | Step 4: Generates MCD | A | A | A | P | P | A | P | A | A | A | A | A | A | A | 19 |
| *modA* | K02020 | Molybdate transporter | P | P | P | P | P | P | P | P | P | A | A | A | A | A | 6 |
| *modB* | K02018 | Molybdate transporter | P | P | P | P | P | P | P | P | P | A | A | A | A | A | 4 |
| *modC* | K02017 | Molybdate transporter | P | P | A | A | A | A | A | A | A | A | A | A | A | A | 5 |
| *modE* | K02019 | Regulation | A | A | A | A | A | A | A | A | A | A | A | A | A | A | 22 |
| *moeA* | K03750 | Step 3: Inserts Mo into MTP | P | P | P | P | P | P | P | P | P | A | A | A | A | A | 103 |
| *moeB* | K21029 | Unknown function | A | A | P | A | A | P | A | P | P | A | A | A | A | A | 41 |
| *mogA* | K03831 | Step 3: Inserts Mo into MTP | A | A | A | A | A | A | A | A | A | A | A | A | A | A | 4 |
| *csrA* | K03563 | Regulation | P | P | P | P | P | P | P | P | P | P | P | P | P | P | 8 |
| *iscS* | K04487 | Sulfur mobilization | P | P | P | P | P | P | P | P | P | P | P | P | P | P | 226 |
| *tusA* | K04085 | Sulfur mobilization | P | P | P | P | P | A | P | P | P | A | A | P | A | P | 99 |

**Table S6.** KEGG functional Orthologs (KO) identifiers associated with heavy metal resistance and tolerance in prokaryotes. The number of copies for each gene identified in the metagenomic dataset SMO1 are indicated in the last column.

| Gene | KO identifier | Definition | Heavy metal | SMO1 copies |
|---|---|---|---|---|
| *arsC* | K03741 | *arsC*; arsenate reductase (thioredoxin) | Arsenic | 113 |
| *arsR* | K03892 | *arsR*; ArsR family transcriptional regulator, arsenate/arsenite/antimonite-responsive transcriptional repressor | Arsenic | 58 |
| *arsB* | K03893 | *arsB*; arsenical pump membrane protein | Arsenic | 6 |
| *zntA* | K01534 | *zntA*; $Zn^{2+}$/$Cd^{2+}$-exporting ATPase | Zinc, cadmium, lead | 1,729 |
| *copA* | K17686 | *copA*, *ctpA*, ATP7; P-type $Cu^{+}$ transporter | Copper | 2,106 |
| *copB* | K01533 | *copB*; P-type $Cu^{2+}$ transporter | Copper | 1,642 |
| *merB* | K00221 | *merB; alkylmercury lyase* | Methylmercury | 0 |

## 2 Supplementary Figures

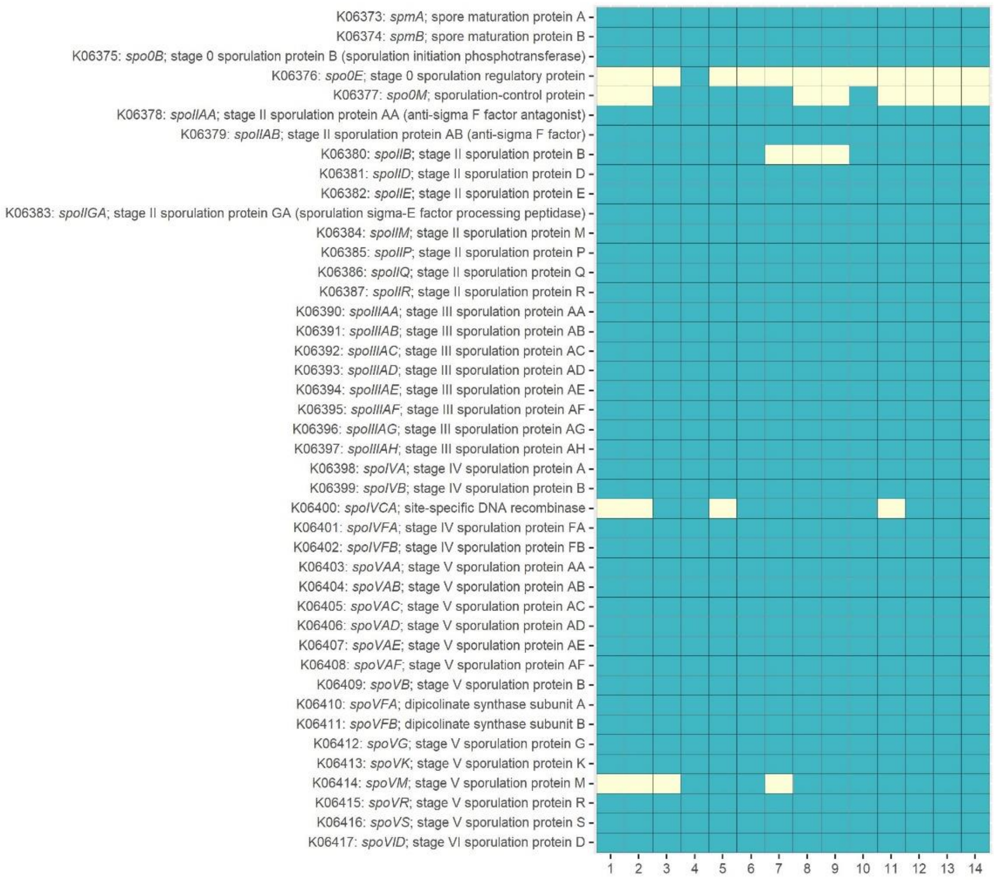


**Supplementary Figure 1.** Heatmap of presence (light blue)/absence (light yellow) of sporulation-related genes in the new strains and closely related species. 1. Strain 3ASR75-11$^{T}$, 2. Strain 3ASR75-286, 3. Strain 3ASR75-54$^{T}$, 4. *A. albus* DSM 23711$^{T}$; 5. *A. halophilus* B6B$^{T}$; 6. *A. kalidii* HU2P27$^{T}$; 7. *A. koreensis* JCM 12387$^{T}$; 8. *A. saliphilus* KHM2$^{T}$; 9. *A. sediminis* BH258$^{T}$; 10. *R. deserti* TKL69$^{T}$; 11. *S. albus* CGMCC 1.6502$^{T}$; 12. *S. dalangtanensis* DP4-553-S$^{T}$; 13. *S. halophilus* CGMCC 1.6199$^{T}$; 14. *S. terrae* JSM 102061$^{T}$.

## REFERENCES


Amoozegar, M. A., Bagheri, M., Didari, M., Mehrshad, M., Schumann, P., Spröer, C., et al. (2014). *Aquibacillus halophilus* gen. nov., sp. nov., a moderately halophilic bacterium from a hypersaline lake, and reclassification of *Virgibacillus koreensis* as *Aquibacillus koreensis* comb. nov. and *Virgibacillus albus* as *Aquibacillus albus* comb. nov. *Int. J. Syst. Evol. Microbiol.* 64, 3616–3623. doi: 10.1099/ijs.0.065375-0.

Carrasco, I. J., Márquez, M. C., Xue, Y., Ma, Y., Cowan, D. A., Jones, B. E., et al. (2008). *Sediminibacillus halophilus* gen.nov., sp. nov., a moderately halophilic, Gram-positive bacterium from a hypersaline lake. *Int. J. Syst. Evol. Microbiol.* 58, 1961–1967. doi: 10.1099/ijs.0.65790-0.

Cho, G.-Y., and Whang, K.-S. (2022). *Aquibacillus saliphilus* sp. nov., a moderately halophilic bacterium isolated from a grey saltern. *Int. J. Syst. Evol. Microbiol.* 72:5496. doi: 10.1099/ijsem.0.005496.

Couto-Rodríguez, R. L. and Montalvo-Rodríguez, R. (2019). Temporal analysis of the microbial community from the crystallizer ponds in Cabo Rojo, Puerto Rico, using metagenomics. *Genes.* 10:422. doi: 10.3390/genes10060422.

Fernández, A. B., Ghai, R., Martin-Cuadrado, A.-B., Sánchez-Porro, C., Rodriguez-Valera, F., and Ventosa, A. (2014a). Prokaryotic taxonomic and metabolic diversity of an intermediate salinity hypersaline habitat assessed by metagenomics. *FEMS Microbiol. Ecol.* 88, 623–635. doi: 10.1111/1574-6941.12329.

Fernández, A. B., Vera-Gargallo, B., Sánchez-Porro, C., Ghai, R., Papke, R. T., Rodriguez-Valera, F., et al. (2014b). Comparison of prokaryotic community structure from Mediterranean and Atlantic saltern concentrator ponds by a metagenomic approach. *Front. Microbiol.* 5:196. doi: 10.3389/fmicb.2014.00196.

Ghai, R., Pašić, L., Fernández, A. B., Martin-Cuadrado, A.-B., Mizuno, C. M., McMahon, K. D., et al. (2011). New abundant microbial groups in aquatic hypersaline environments. *Sci. Rep.* 1:135. doi: 10.1038/srep00135.

Kheiri, R., Mehrshad, M., Pourbabaee A. A., Ventosa, A., and Amoozegar, M. A. (2023). Hypersaline Lake Urmia: a potential hotspot for microbial genomic variation. *Sci. Rep.* 13:374. doi: 10.1038/s41598-023-27429-2.

Lee, J.-C., and Whang, K.-S. (2019). *Aquibacillus sediminis* sp. nov., a moderately halophilic bacterium isolated from saltern soil. *Int. J. Syst. Evol. Microbiol.* 69, 3121–3127. doi: 10.1099/ijsem.0.003599.

Lee, J.-S., Lim, J.-M., Lee, K.-C., Lee, J.-C., Park, Y.-H., and Kim, C.-J. (2006). *Virgibacillus koreensis* sp. nov., a novel bacterium from a salt field, and transfer of *Virgibacillus picturae* to the genus *Oceanobacillus* as *Oceanobacillus picturae* comb. nov. with emended descriptions. *Int. J. Syst. Evol. Microbiol.* 56, 251–257. doi: 10.1099/ijs.0.63734-0.

Li, J., Zhang, B., Liu, G., Liu, Y., Yang, H., Yang, R., et al. (2020). *Radiobacillus deserti* gen. nov., sp. nov., a UV-resistant bacterium isolated from desert soil. *Int. J. Syst. Evol. Microbiol.* 70, 6338–6347. doi: 10.1099/ijsem.0.004536.

Magnuson, E., Altshuler, I., Fernández-Martínez, M. Á., Chen, Y.-J., Maggiori, C., Goordial, J., et al. (2022). Active lithoautotrophic and methane-oxidizing microbial community in an anoxic, sub-zero, and hypersaline High Arctic spring. *ISME J.* 16, 1798-1808. doi: 10.1038/s41396-022-01233-8.

Patel, R., Mevada, V., Prajapati, D., Dudhagara, P., Koringa, P., and Joshi, C. G. (2015). Metagenomic sequence of saline desert microbiota from wild ass sanctuary, Little Rann of Kutch, Gujarat, India. *Genom. Data.* 3, 137–139. doi: 10.1016/j.gdata.2015.01.003.

Perez, M. F., Kurth, D., Farías, M. E., Soria, M. N., Castillo Villamizar, G. A., Poehlein, A., et al. (2020). First report on the plasmidome from a high-altitude lake of the Andean Puna. *Front. Microbiol*. 11:1343. doi: 10.3389/fmicb.2020.01343.

Plominsky, A. M., Delherbe, N., Ugalde, J. A., Allen, E. E., Blanchet, M., Ikeda, P., et al. (2014). Metagenome sequencing of the microbial community of a solar saltern crystallizer pond at Cáhuil Lagoon, Chile. *Genome Announc.* 2, e01172–e01114. doi: 10.1128/genomeA.01172-14.

Podell, S., Emerson, J. B., Jones, C. M., Ugalde, J. A., Welch, S., Heidelberg, K. B., et al. (2014). Seasonal fluctuations in ionic concentrations drive microbial succession in a hypersaline lake community. *ISME J.* 8, 979–990. doi: 10.1038/ismej.2013.221.

Vera-Gargallo, B., and Ventosa, A. (2018). Metagenomic insights into the phylogenetic and metabolic diversity of the prokaryotic community dwelling in hypersaline soils from the Odiel Saltmarshes (SW Spain). *Genes (Basel)* 9:152. doi: 10.3390/genes9030152.

Wang, H. T., Xu, L., and Sun, J. Q. (2021). *Aquibacillus kalidii* sp. nov., an indole acetic acid-producing endophyte from a shoot of *Kalidium cuspidatum,* and reclassification of *Virgibacillus campisalis* Lee et al. 2012 as a later heterotypic synonym of *Virgibacillus alimentarius* Kim et al. 2011. *Int. J. Syst. Evol. Microbiol.* 71:5030. doi: 10.1099/ijsem.0.005030.

Xie, Y. G., Luo, Z. H., Fang, B. Z., Jiao, J. Y., Xie, Q. J., Cao, X. R., et al. (2022) Functional differentiation determines the molecular basis of the symbiotic lifestyle of *Ca.* Nanohaloarchaeota. *Microbiome* 10: 172. doi: 10.1186/s40168-022-01376-y.

Zhang, W.-Y., Hu, J., Zhang, X.-Q., Zhu, X.-F., and Wu, M. (2015). *Aquibacillus salifodinae* sp. nov., a novel bacterium isolated from a salt mine in Xinjiang province, China. *Antonie van Leeuwenhoek* 107, 367–374. doi: 10.1007/s10482-014-0335-9.

Zhang, Y. J., Zhou, Y., Ja, M., Shi, R., Chun-Yu, W. X., Yang, L. L., et al. (2012). *Virgibacillus albus* sp. nov., a novel moderately halophilic bacterium isolated from Lop Nur salt lake in Xinjiang province, China. *Antonie van Leeuwenhoek* 102, 553–560. doi: 10.1007/s10482-012-9750-y.
